# Supplementary figures and images for: Enterococcal cell wall remodelling underpins pathogenesis via the release of the Enteroccocal Polysaccharide Antigen (EPA)
Source: PLoS Pathog. 2025 Jun 23;21(6):e1012771. doi: 10.1371/journal.ppat.1012771 (PMC12208459; doi:10.1371/journal.ppat.1012771)

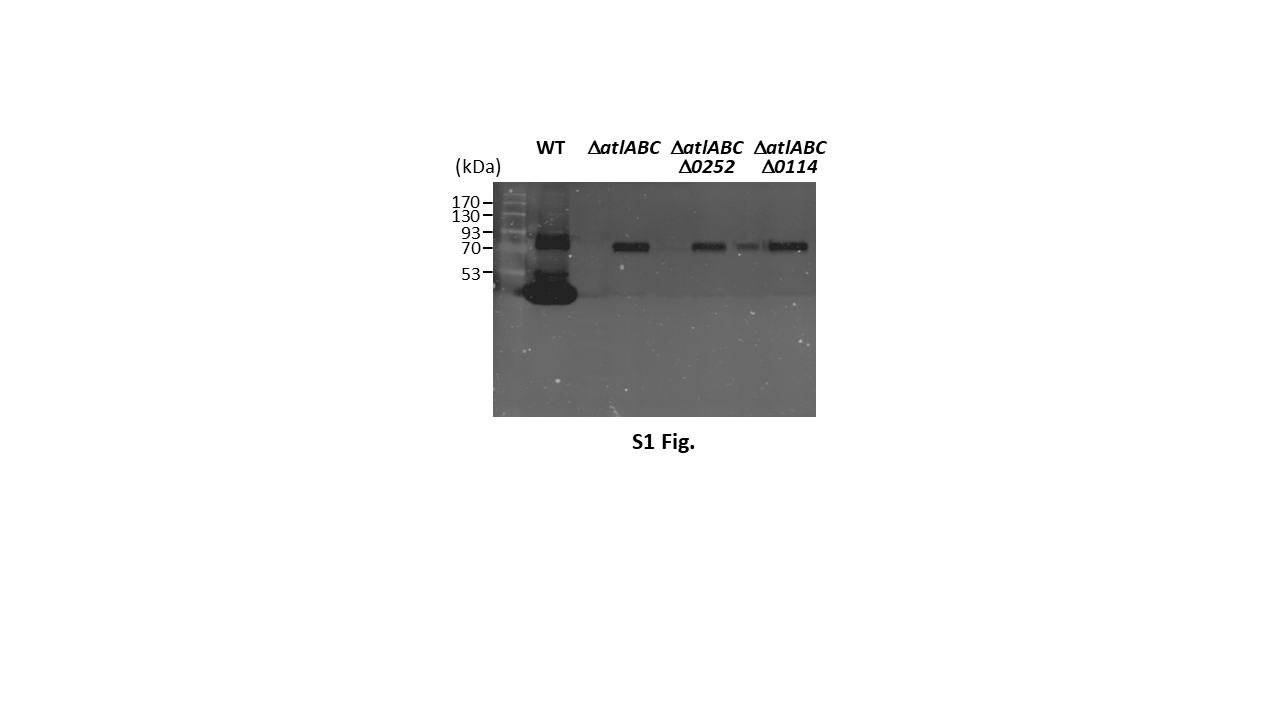

Supplement: S1 Fig — Peptidoglycan hydrolytic activities were detected in 20 µL of culture supernatants of strains JH2–2 (WT), ΔatlABC, ΔatlABC Δ0252 and ΔatlABC Δ0114 grown overnight. Cells from the triple class A PBP mutant ΔponA ΔpbpF ΔpbpZ were used as a substrate and zymograms were incubated for 72h at 37°C. (TIF) [file ppat.1012771.s001.tif]

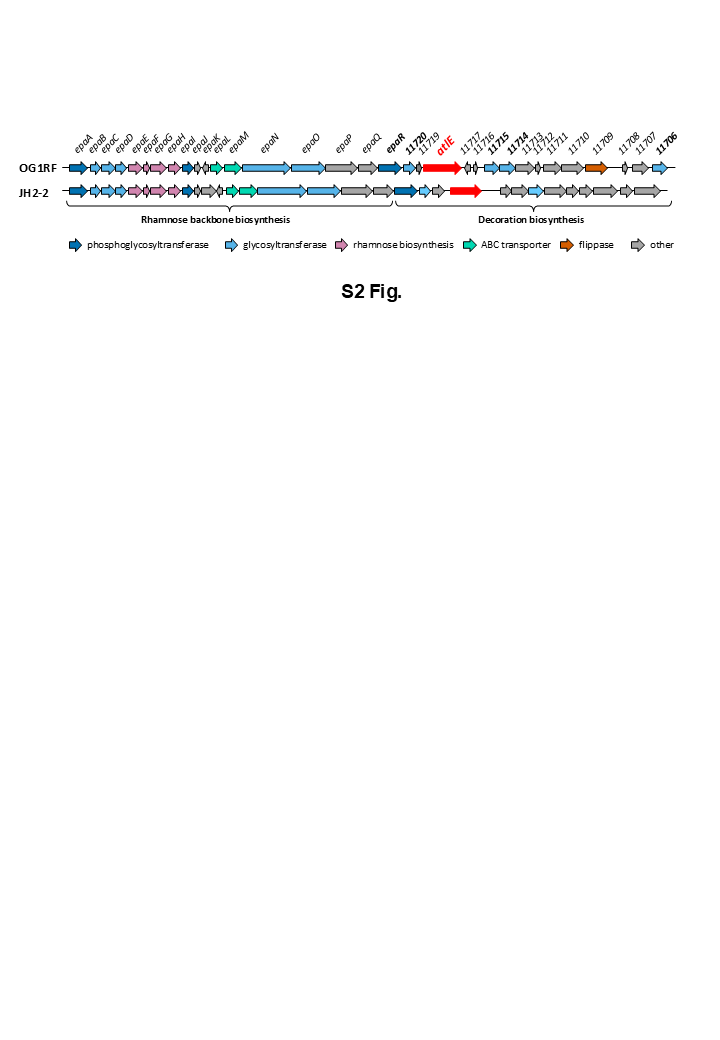

Supplement: S2 Fig — Genes epaA to epaR are conserved across strains epaR to 11706 encode EPA decorations which can vary between strains. (TIF) [file ppat.1012771.s002.tif]

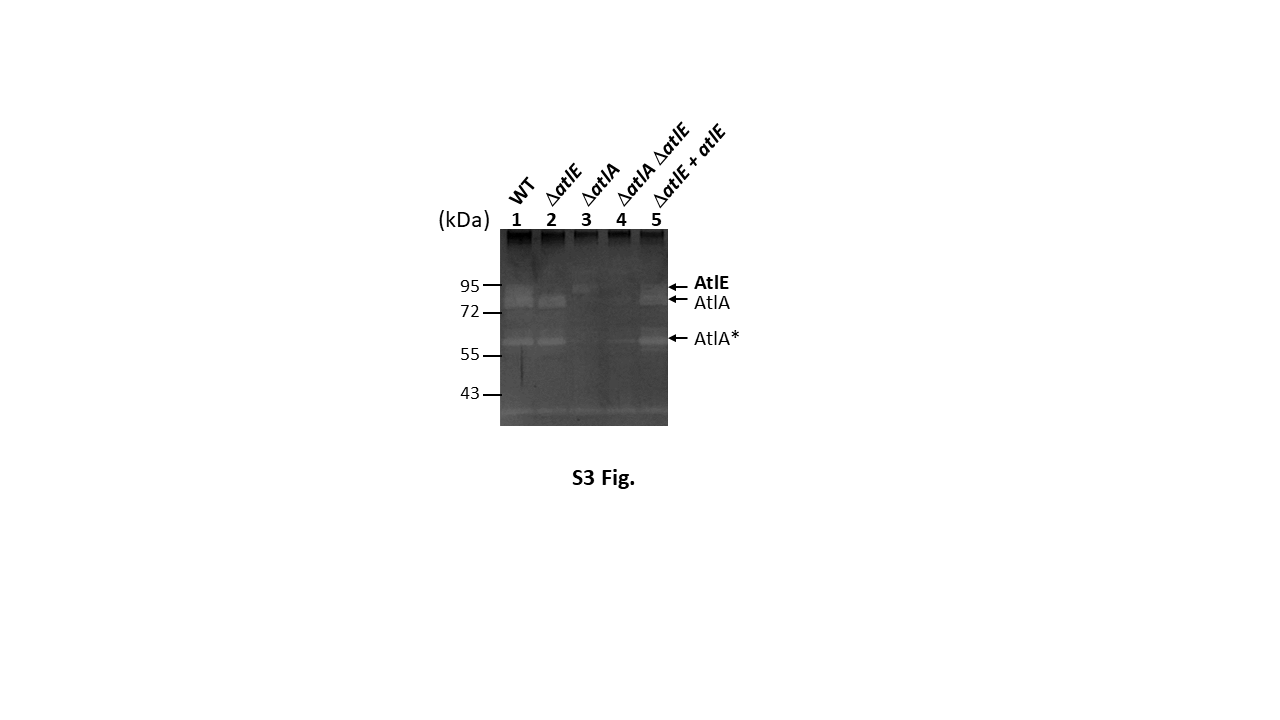

Supplement: S3 Fig — Peptidoglycan hydrolytic activities were detected in 25 µL of culture supernatants of strains OG1RF (WT, lane 1), ΔatlE (lane 2), ΔatlA (lane 3), ΔatlA ΔatlE (lane 4), and complemented ΔatlA ΔatlE mutant (ΔatlE + atlE, lane 5). Cells from the triple class A PBP mutant ΔponA ΔpbpF ΔpbpZ were used as a substrate. (TIF) [file ppat.1012771.s003.tif]

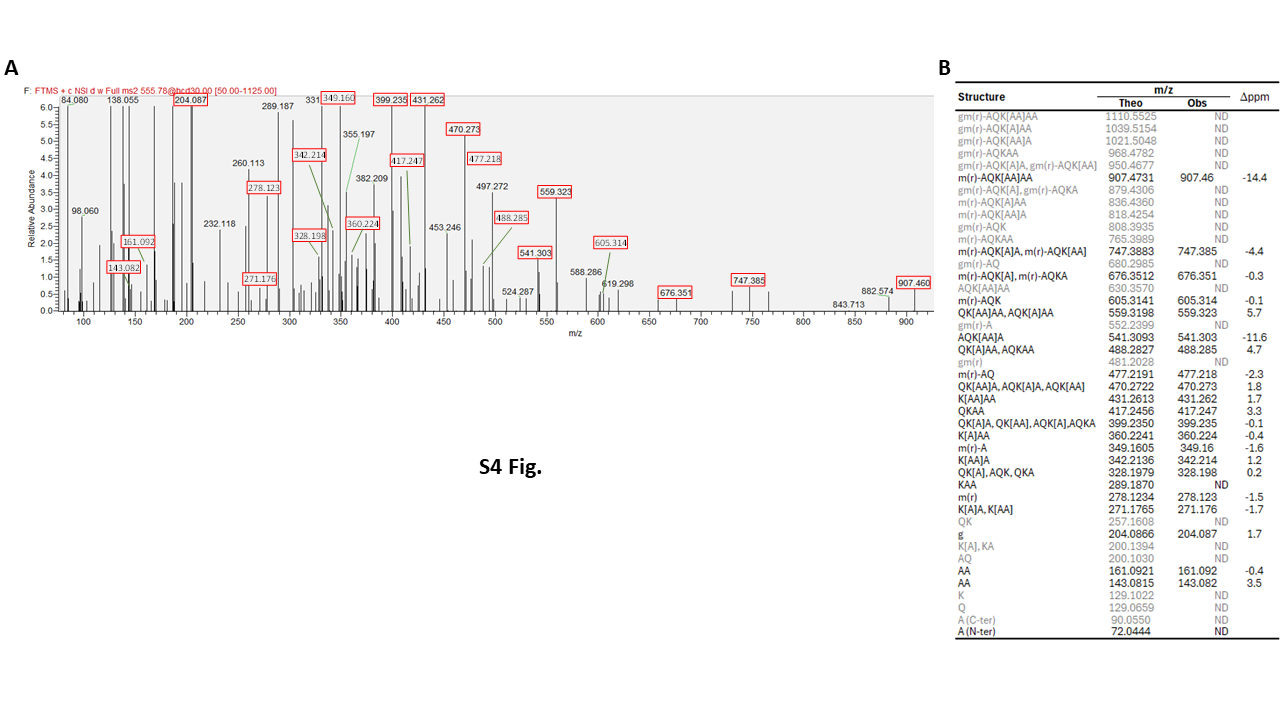

Supplement: S4 Fig — A, fragmentation of the doubly charged Ion (M + 2H]2+; m/z = 555.78). Ions with m/z values matching predicted fragments are boxed in red. B, List of predicted fragments, theoretical and observed m/z. ND, not detected; g, GlcNAc; m(r), reduced MurNAc; residues in square bracket correspond to the lateral chain. (TIF) [file ppat.1012771.s004.tif]

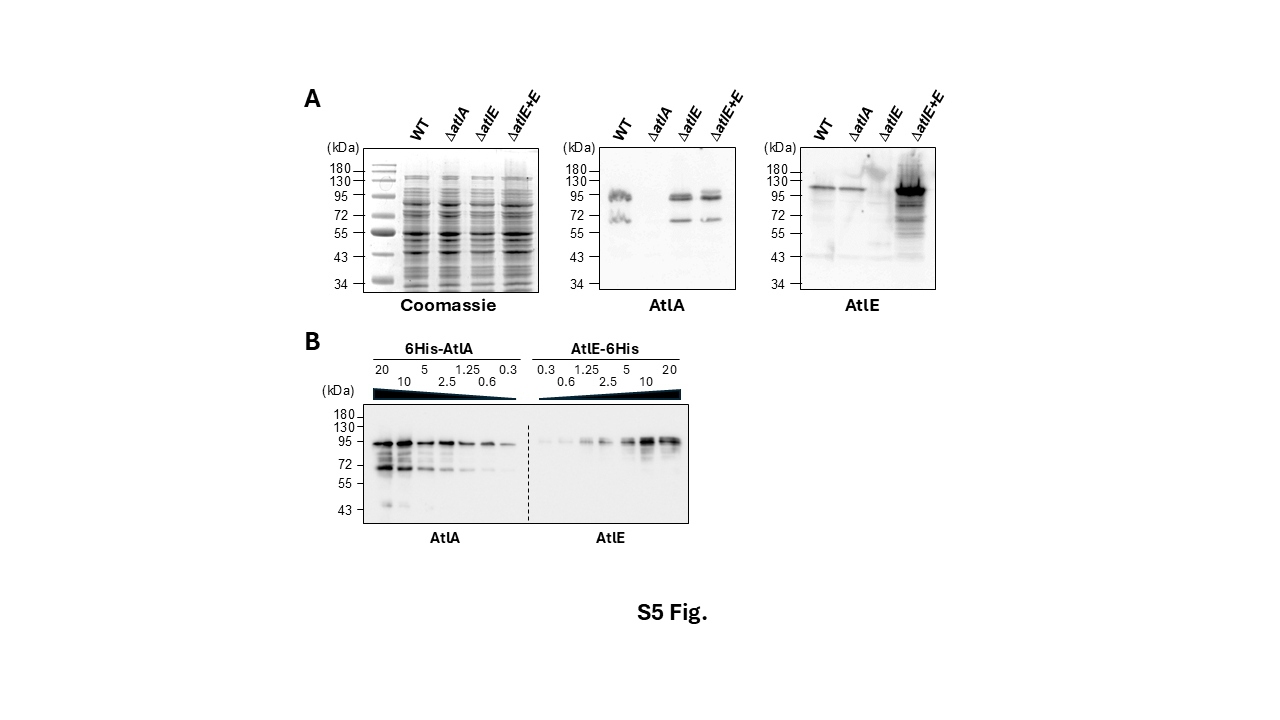

Supplement: S5 Fig — A, Specificity of antibodies raised against recombinant AtlA and AtlE proteins was tested against E. faecalis crude extracts from cells grown in exponential phase (OD600nm≈0.3); WT is OG1RF, ΔatlE + E corresponds to the ΔatlE mutant complemented. For AtlA detection, 2 µg of crude extracts were used; primary serum was used at a dilution of 1/25,000. For AtlE detection, 5µg of crude extracts were used; primary serum was used at a dilution of 1/10,000. In both cases, secondary antibodies (goat anti-rabbit antibodies coupled to horseradish peroxidase) were used at a 1/20,000 dilution. B, sensitivity of anti-AtlA and anti-AtlE antibodies. (TIF) [file ppat.1012771.s005.tif]

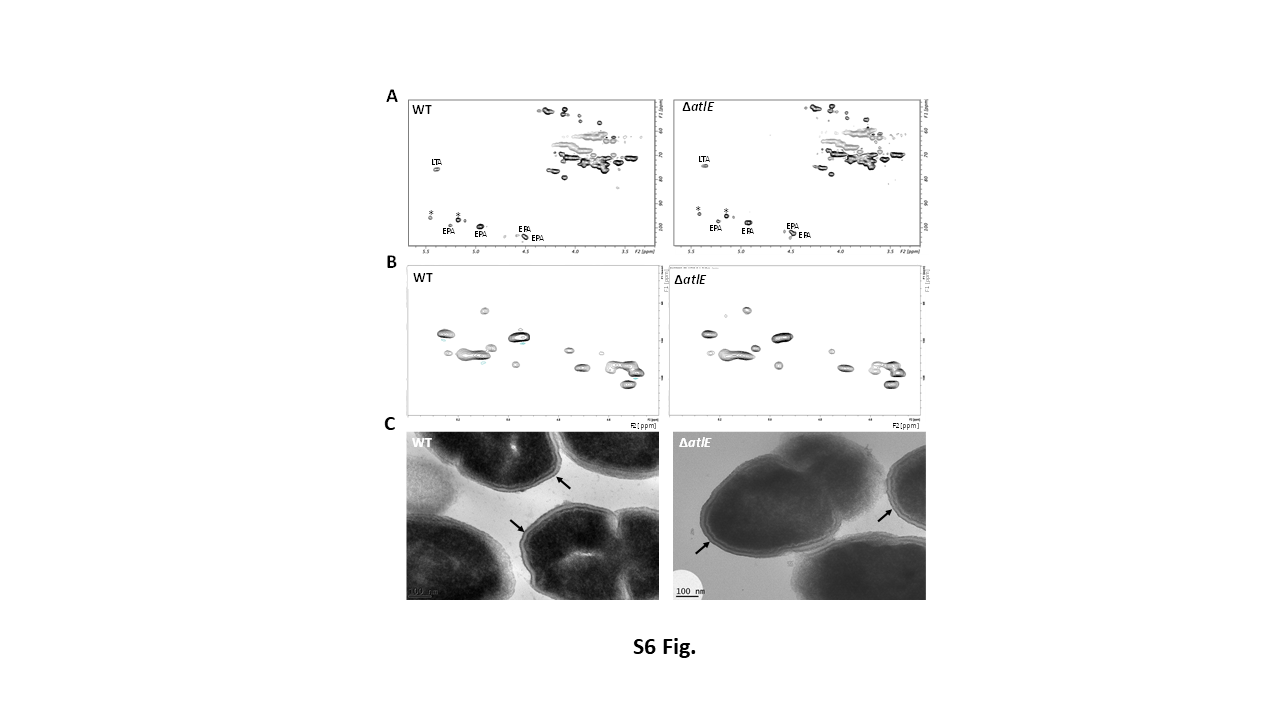

Supplement: S6 Fig — A, 1H-13C HSQC spectra of purified wild type and ΔatlE EPA. The region displayed corresponding to the anomeric protons (4.3-5.5 ppm) and anomeric carbons (90–110 ppm) of WT (left) and ΔatlE (right) did not reveal any major difference between the 2 EPA polymers. B, 1H-13C HSQC HR-MAS NMR experiments recorded on E. faecalis OG1RF (left) and ΔatlE (right) cells show that AtlE does not contribute towards the production or display of surface exposed EPA or lipoteichoic acid (LTA) [26]. Two other currently unidentified cell wall polysaccharides denoted with an asterisk are also detected [21]. C, Thin section transmission electron microscopy of E. faecalis OG1RF (left) and ΔatlE (right) cells confirm EPA decorations remain surface exposed in the ΔatlE mutant, both forming a pellicle at their cell surface (arrows). (TIF) [file ppat.1012771.s006.tif]

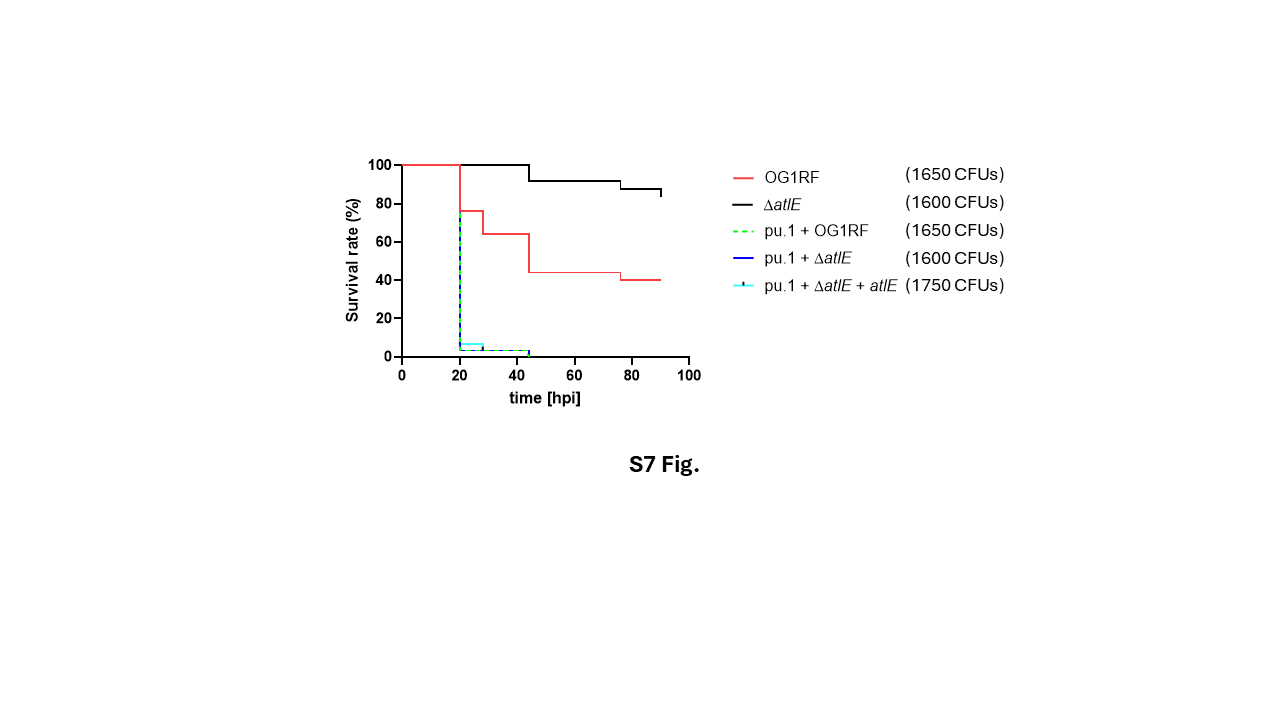

Supplement: S7 Fig — Larvae were infected with ca. 1,650 CFUs of parental OG1RF strain (solid red line) or ΔatlE (solid black line). Phagocyte depletion was performed using pu.1 morpholinos before injection with OG1RF (green dashed line), ΔatlE (solid blue line) or ΔatlE + atlE cells (green solid line). Survival was monitored between 20–90 hours post infection (hpi) at 28°C using 25 larvae per strain. (TIF) [file ppat.1012771.s007.tif]

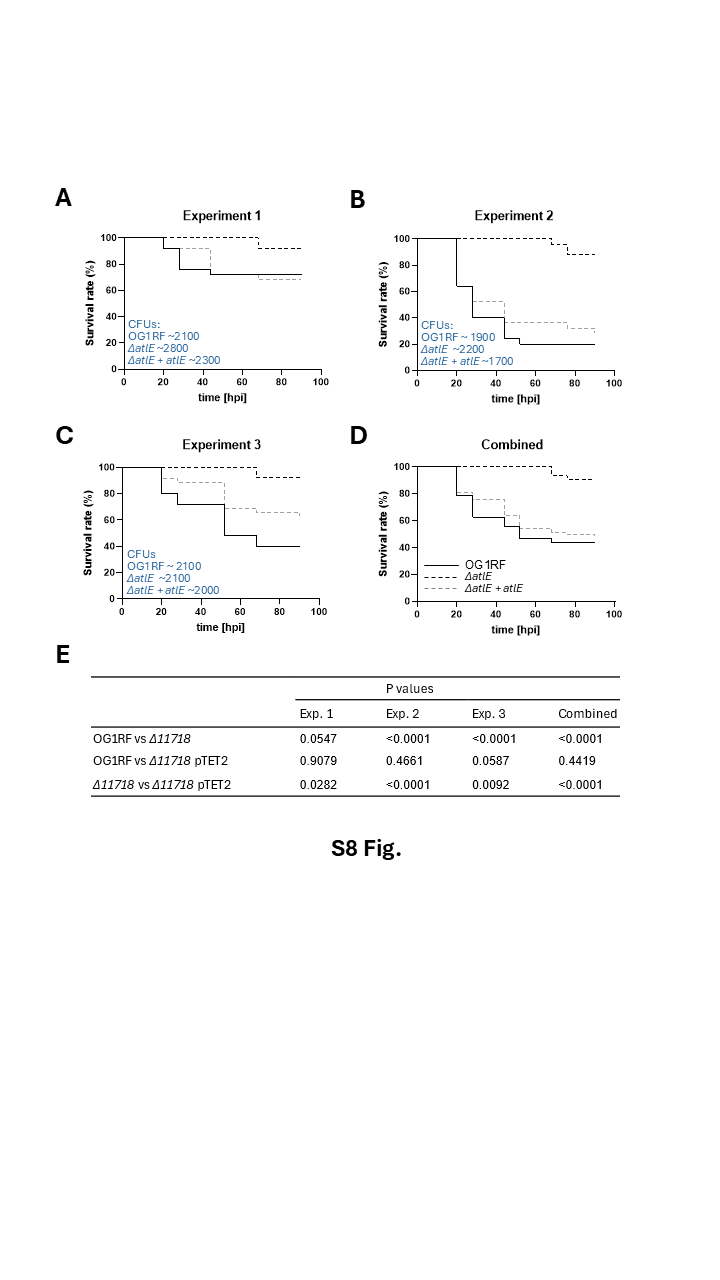

Supplement: S8 Fig — Larvae were infected with ca. 2,000 CFUs of parental (WT) OG1RF strain (solid line), ΔatlE (black dashed line) or ΔatlE + atlE (grey dashed line). Survival was monitored between 20–90 hours post infection (hpi) at 28°C using 25 larvae per strain per experiment. Three independent experiments (A, B and C) and combined results (D) are shown. (E) P values of pairwise comparison. (TIF) [file ppat.1012771.s008.tif]

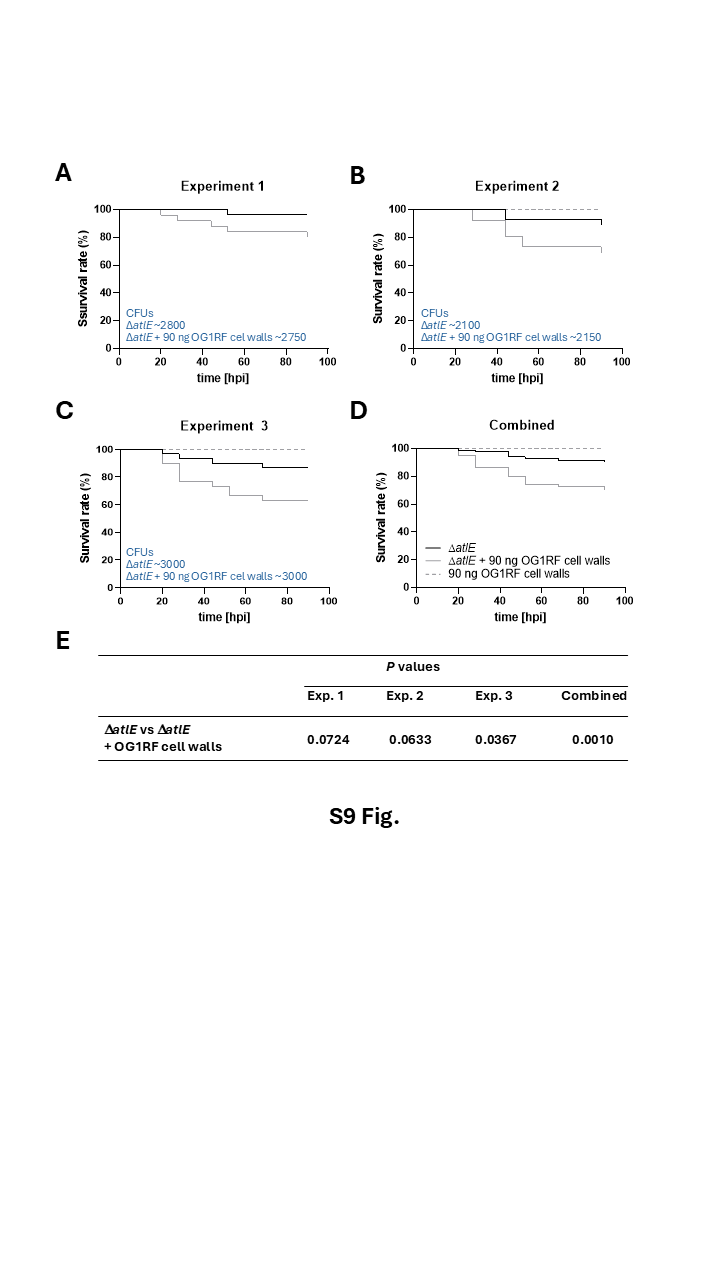

Supplement: S9 Fig — Larvae were infected with ca. 2,000 CFUs of the ΔatlE strain in the absence (solid line) or presence (grey line) of 90 ng of soluble cell walls. A control injection corresponding to 90 ng of OG1RF cell walls alone is shown (grey dashed line). Survival was monitored between 20–90 hours post infection (hpi) at 28°C using at least 25 larvae per strain per experiment. Three independent experiments (A, B and C) and combined results (D) are shown. E, P values of pairwise comparisons. (TIF) [file ppat.1012771.s009.tif]

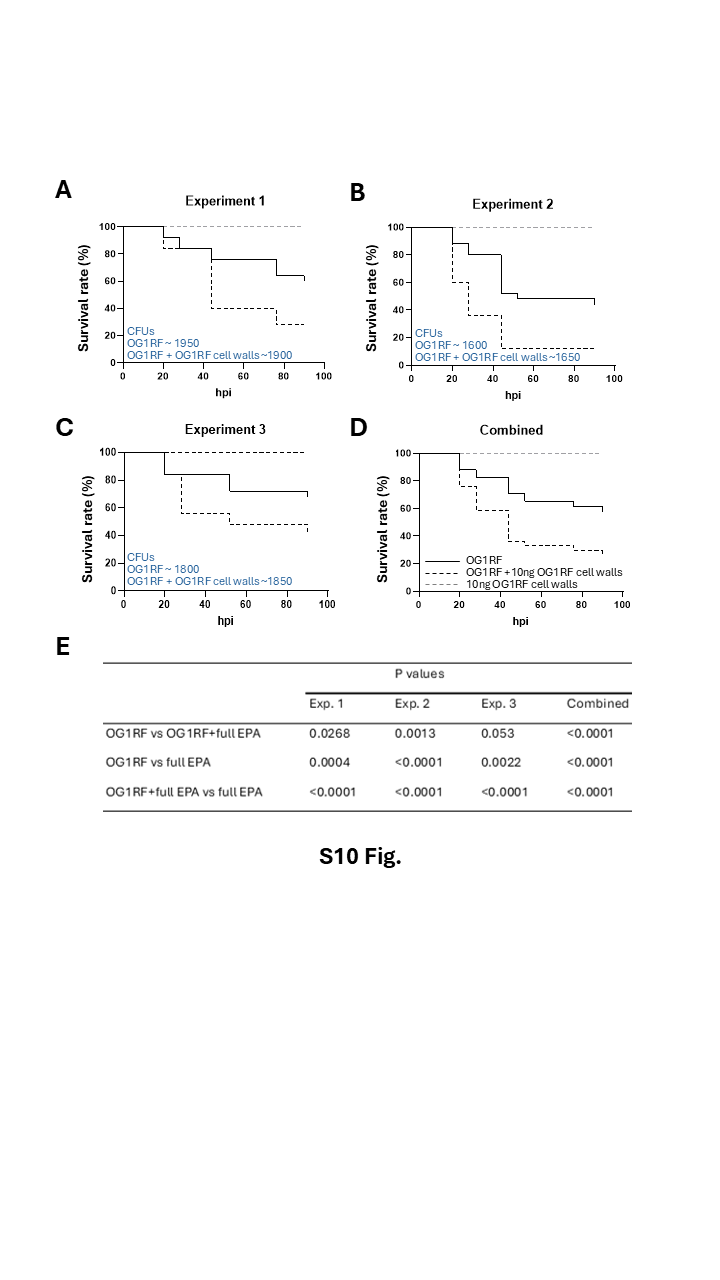

Supplement: S10 Fig — Larvae were infected with ca. 2,000 CFUs of parental (WT) OG1RF strain in the absence (solid line) or presence (black dashed line) of 10 ng of soluble cell walls. A control injection corresponding to 10ng of OG1RF cell walls alone is shown (grey dashed line). Survival was monitored between 20–90 hours post infection (hpi) at 28°C using 25 larvae per strain per experiment. Three independent experiments (A, B and C) and combined results (D) are shown. (E) P values of pairwise comparison. (TIF) [file ppat.1012771.s010.tif]

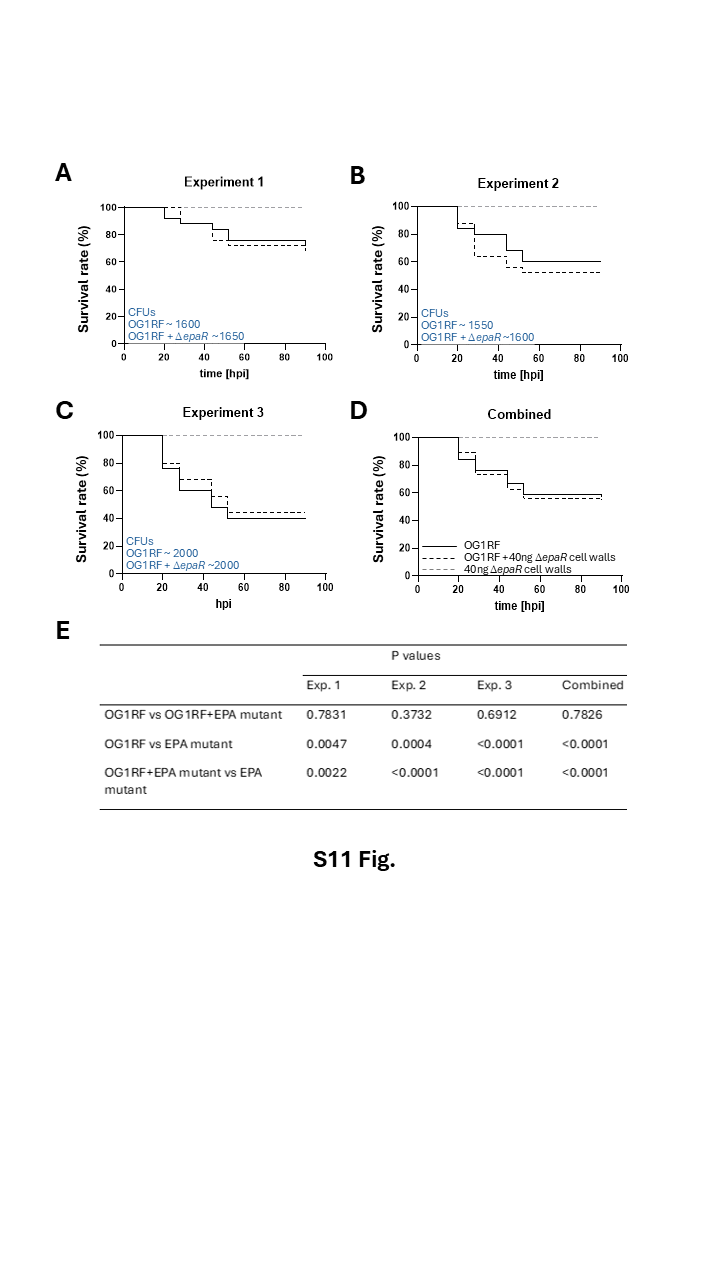

Supplement: S11 Fig — Larvae were infected with ca. 2,000 CFUs of parental (WT) OG1RF strain in the absence (solid line) or presence (black dashed line) of 40 ng of soluble cell walls. A control injection corresponding to 40 ng of OG1RF cell walls alone is shown (grey dashed line). Survival was monitored between 20–90 hours post infection (hpi) at 28°C using 25 larvae per strain per experiment. Three independent experiments (A, B and C) and combined results (D) are shown. (E) P values of pairwise comparison. (TIF) [file ppat.1012771.s011.tif]

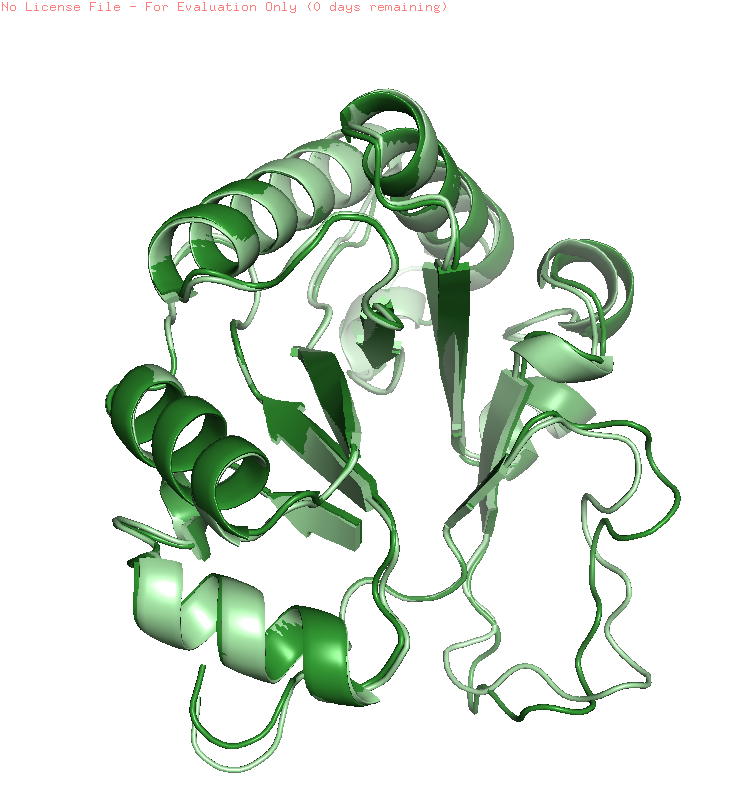

Supplement: S1 File — Zipped raw data used to make figures 1–10 are provided in individual folders for each figure. (ZIP) [file ppat.1012771.s013.zip › Data Smith et al/Fig. 4/GH25 J and O colourblind.png]

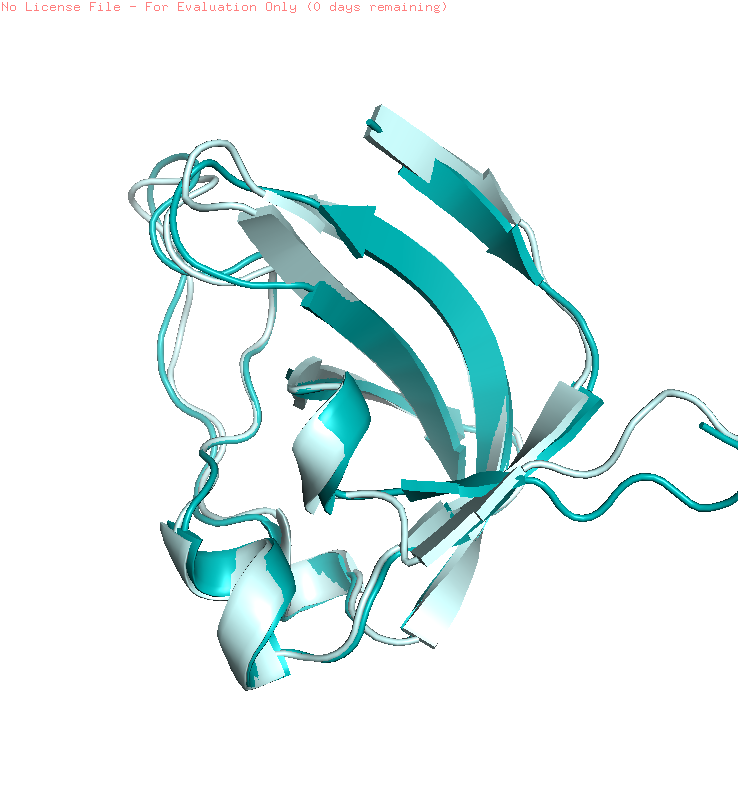

Supplement: S1 File — Zipped raw data used to make figures 1–10 are provided in individual folders for each figure. (ZIP) [file ppat.1012771.s013.zip › Data Smith et al/Fig. 4/R1_J R2_J colourblind.png]

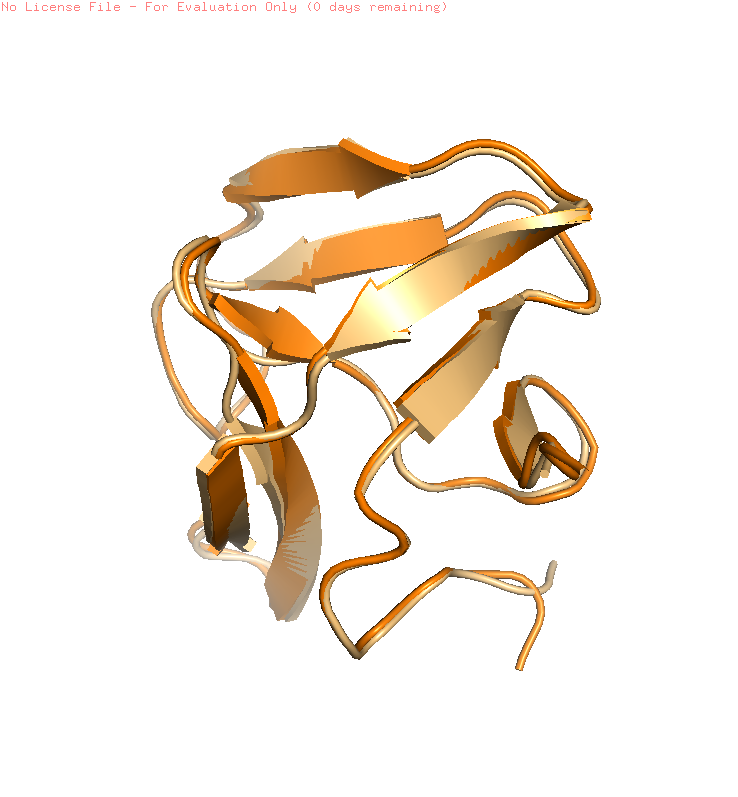

Supplement: S1 File — Zipped raw data used to make figures 1–10 are provided in individual folders for each figure. (ZIP) [file ppat.1012771.s013.zip › Data Smith et al/Fig. 4/R1_O R6_O colourblind.png]

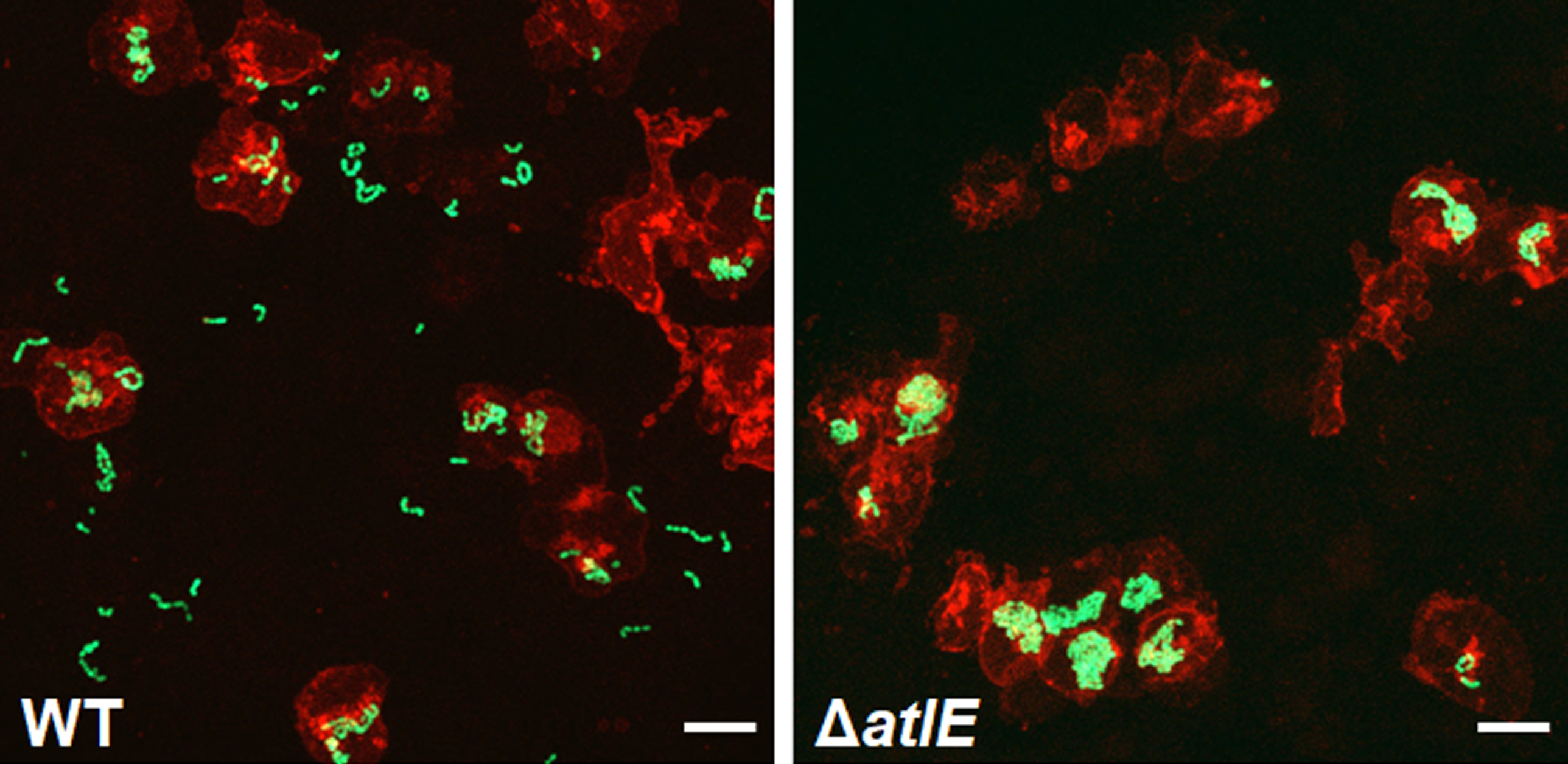

Supplement: S1 Striking Fig — Zebrafish embryos of the Tg(mpeg:mCherry-F) transgenic line were infected with 2,000 CFUs of E. faecalis cells constitutively producing GFP. Representative images show E. faecalis uptake in zebrafish embryos 1.5 h post infection with the WT strain (OG1RF) and a ΔatlE derivative. Phagocytes labeled with mCherry appear in red, and GFP-labelled bacteria in green. Scale bar is 10 μm. Robert E Smith, Bartosz J Michno, Rene L Christena, Finn O’Dea, Jessica L Davis, Ian D.E.A. Lidbury, Marcel G Alamán-Zárate, Danai Stefanidi, Emmanuel Maes, Hannah Fisher, Tomasz K Prajsnar and Stéphane Mesnage. Enterococcal cell wall remodelling underpins pathogenesis via the release of the Enteroccocal Polysaccharide Antigen (EPA). This image ca be can publish under the Creative Commons Attribution License (https://creativecommons.org/licenses/by/4.0/). (JPG) [file ppat.1012771.s017.jpg]
