# Supplementary material for: Enterococcal cell wall remodelling underpins pathogenesis via the release of the Enteroccocal Polysaccharide Antigen (EPA)
Source: PLoS Pathog. 2025 Jun 23;21(6):e1012771. doi: 10.1371/journal.ppat.1012771 (PMC12208459; doi:10.1371/journal.ppat.1012771)
Supplement: S1 Table — (DOCX) [file ppat.1012771.s012.docx]

**S1 Table.** Bacterial strains, plasmids, and oligonucleotides.

| **Strains/plasmids/**  **oligonucleotides** | **Relevant properties/sequence** | | **Source** | | |  |
| --- | --- | --- | --- | --- | --- | --- |
| **Strains** |  | | |  | |  |
| *Enterococcus faecalis* |  | | |  | |  |
| JH2-2 | Laboratory strain | | | | (1) | |
| JH2-2 Δ*atlABC* | JH2-2 derivative with an in-frame deletions in *atlA, atlB* and *atlC* | | | | (2) | |
| JH2-2 Δ*atlABC* Δ*0114* | JH2-2 Δ*atlABC* derivative with an in-frame deletions in *TX4000_00147* | | | | This work | |
| JH2-2 Δ*atlABC* Δ*0252* | JH2-2 Δ*atlABC* derivative with an in-frame deletions in *TX4000_00259* | | | | This work | |
| JH2-2 Δ*atlABC* Δ*atlE* | JH2-2 Δ*atlABC* derivative with an in-frame deletions in *TX4000_01873* | | | | This work | |
| JH2-2 Δ*pbp* | JH2-2 Δ*ponA* Δ*pbpF* Δ*pbpZ*; substrate for zymogram experiments | | | | (3) | |
| OG1RF | Clinical isolate from human oral cavity | | | | (4) | |
| OG1RF Δ*atlA* | OG1RF derivative with an in-frame deletion in *atlA* | | | | (5) | |
| OG1RF Δ*atlE* | OG1RF derivative with an in-frame deletion in *OG1RF_11718* | | | | This work | |
| OG1RF Δ*atlA* Δ*atlE* | Double *atlA atlE* mutant | | | | This work | |
| OG1RF Δ*11720* | OG1RF derivative with an in-frame deletion in *OG1RF_11720* | | | | (6) | |
| OG1RF Δ*11720+11720* | OG1RF Δ*11720* complemented strain | | | | (6) | |
| *Escherichia coli* |  | | | |  | |
| NEB5alpha | Cloning strain | | | | NEB | |
| BL21(DE3) | Protein expression strain | | | | NEB | |
| **Plasmids** |  | | | |  | |
| pGhost9 | Plasmid for allelic replacement in *E. faecalis* (Erm^R^) | | | | (7) | |
| pTetH | Plasmid for complementation (anhydrotetracyline-induced expression) (Erm^R^) | | | | (5) | |
| pET2818 | pET derivative for protein production (C-terminal histidine-tag) (Amp^R^) | | | | (8) | |
| pGHH0252 | pGhost9 derivative for *EF0252* deletion | | | | This work | |
| pGHH0114 | pGhost9 derivative for *EF0114* deletion | | | | This work | |
| pGHH_atlE_J | pGhost9 derivative for *atlE* deletion in JH2-2 | | | | This work | |
| pGHH_atlE_O | pGhost9 derivative for *atlE* deletion in OG1RF | | | | This work | |
| pET_AtlE_O | pET2818 derivative encoding AtlE allele from OG1RF (residues 25 to 818) (Amp^R^) | | | | This work | |
| pTet_AtlE_J | pTetH derivative encoding full length AtlE from JH2-2 for complementation (Amp^R^) | | | | This work | |
| pTet_AtlE_O | pTetH derivative encoding full length AtlE from OG1RF for complementation (Amp^R^) | | | | This work | |
| **Oligonucleotides** |  | | | |  | |
| EF0252_H11 | TATAGGGCGAATTGGGTACCGGGCCCCCCCTCGAGAACCTTTAGAAGGAATTGAACggaaaatt |  | | | |  |
| EF0252_H12 | GTTATTACCTTCTGCAAATGCACCTACTGGC |  | | | |  |
| EF0252_H21 | GGTGCATTTGCAGAAGGTAATAACAAGGGATTAAACGTTGTTTCGACACGTA |  | | | |  |
| EF0252_H22 | CTCTAGCTAGTGGATCCCCCGGGCTGCAGGAATTCGCGTTTCCCGAAGCGGTTTTCC |  | | | |  |
| EF0252_H110 | TCTATTACGGGCGACAGGGGTCG |  | | | |  |
| EF0252_H220 | GGTCTGCACTGGCAGGGACATCAAT |  | | | |  |
| EF0114_H11 | TATAGGGCGAATTGGGTACCGGGCCCCCCCTCGAGCAGCCAGGCCAGAAAGTCCTGATT |  | | | |  |
| EF0114_H12 | TGCCAAGCCAACCAATAACGAAAGACC |  | | | |  |
| EF0114_H21 | GTCTTTCGTTATTGGTTGGCTTGGCACCAATTATTGGAAGTATTCAATGTGTTTGG |  | | | |  |
| EF0114_H22 | CTCTAGCTAGTGGATCCCCCGGGCTGCAGGAATTCAAGTGTCTCCATTTGAACAGAAGC |  | | | |  |
| EF0114_H110 | GTCGGTTTGACAAGTTATCAAGCGC |  | | | |  |
| EF0114_H220 | CCAGCTTGAGGTGCATTAGGGATAG |  | | | |  |
| atlE_H11_J | aaaCTCGAGTTATTAGGGATTTTTTCTTCAAGCAAAATTCGCT |  | | | |  |
| atlE_H12_J | CACTATTGATGAACTAGGAACCAATGCTGT |  | | | |  |
| atlE_H21_J | TTCCTAGTTCATCAATAGTGGCTGGGTAGATAGTCGAGCATTAAAAAAATAAC |  | | | |  |
| atlE_H22_J | CAGGAATTCCCTGTCCATAATACATTCTTTAACAAACCCTACC |  | | | |  |
| atlE_H11_O | aaaCTCGAGAACCACCTCTACATCTTCTAACAAATGG |  | | | |  |
| atlE_H12_O | TTTATTTGAAGTGATATACCCTTGTGATGCTTGAGTGACCAAGCCAG  CAACGGGGGAGACTGGCTTGGTCACTCAAGCATCACAAGGGTATATCACTTCA |  | | | |  |
| atlE_H21_O | CAACGGGGGAGACTGGCTTGGTCACTCAAGCATCACAAGGGTATATCACTTCA  CCCCGGGCTGCAGGAATTCGATATCAAGCTTCATTCACCCCTGCTCTTAC |  | | | |  |
| atlE_H22_O | CAGGAATTCGATATCAAGCTTCATTCACCCCTGCTCTTAC |  | | | |  |
| atlE_H110 | TTTTATGCCACGACCAGATTATG |  | | | |  |
| atlE_H220 | AAATTGTTTAGGAATCTCCTGCC |  | | | |  |
| atlE_J_Fw | Cgtgagctcaaggaggagactgaccatgaagagaataaataaaatatctgttattacaatgctaa |  | | | |  |
| atlE_J_Rev | gtgggatccttatttttttaatgctcgactatctacccagcc |  | | | |  |
| atlE_O_Fw | tctgagctcAAGGAGGAGACTGACCatgaaaaaaatcatttcaggtatgtt |  | | | |  |
| atlE_O_Rev | gcggGAtcCTTAATTCACTTTTTGTACATAACGTt |  | | | |  |
| atlE_O_pETF | CCCTCTAGAAATACTTTTGTTTAACTTTAAGAAGGAGATATACGATGGAAGAGCTTGTAAAAACAGAAACAAC |  | | | |  |
| atlE_O_pETR | ATGGGATCCATTCACTTTTTGTACATAACGTTTATTTGAAGTGAT |  | | | |  |
| pTetH_Fw | GCTTGATCGTAGCGTTAACAGATCTACTC |  | | | |  |
| pTetH_Rev | CAAATTGTGGATGTGACCATGCGG |  | | | |  |
| pGhost_Fw | GTCACGACGTTGTAAAACGACGG |  | | | |  |
| pGhost_Rev | CTAGCGGACTCTAGAGGATCCCA |  | | | |  |

Erm^R^, resistance to erythromycin; Amp^R^, resistance to ampicillin; restriction sites are highlighted in yellow

**References**

1. Jacob AE, and Hobbs SJ. 1974. Conjugal transfer of plasmid-borne multiple antibiotic resistance in *Streptococcus faecalis* var. *zymogenes*. *J Bacteriol,* 117**:** 360-372.
2. Mesnage S, Chau F, Dubost L, Arthur M. 2008. Role of *N*-acetylglucosaminidase and *N*-acetylmuramidase activities in *Enterococcus faecalis* peptidoglycan metabolism. *J Biol Chem*, 283(28): 19845-53.
3. Arbeloa A, Segal H, Hugonnet JE, Josseaume N, Dubost L, Brouard JP, Gutmann L, Mengin-Lecreulx D, Arthur M. 2004. Role of class A penicillin-binding proteins in PBP5-mediated beta-lactam resistance in *Enterococcus faecalis*. *J Bacteriol,* 186, 1221–1228.
4. Dunny GM, Brown BL and Clewell DB. 1978. Induced cell aggregation and mating in *Streptococcus faecalis*: evidence for a bacterial sex pheromone. *Proc Natl Acad Sci USA,* 75: 3479-3483.
5. Salamaga B, Prajsnar TK, Jareño-Martinez A, Willemse J, Bewley MA, Chau F, Ben Belkacem T, Meijer AH, Dockrell DH, Renshaw SA, Mesnage S. 2017. Bacterial size matters: Multiple mechanisms controlling septum cleavage and diplococcus formation are critical for the virulence of the opportunistic pathogen *Enterococcus faecalis*. *PLoS Pathog*, 13(7):e1006526.
6. Smith RE, Salamaga B, Szkuta P, Hajdamowicz N, Prajsnar TK, Bulmer GS, Fontaine T, Kołodziejczyk J, Herry JM, Hounslow AM, Williamson MP, Serror P, Mesnage S. Decoration of the enterococcal polysaccharide antigen EPA is essential for virulence, cell surface charge and interaction with effectors of the innate immune system. PLoS Pathog. 2019 May 2;15(5):e1007730.
7. Maguin E, Duwat P, Hege T, Ehrlich D & Gruss A. 1992. New thermosensitive plasmid for gram-positive bacteria. *J Bacteriol,* 174: 5633-5638.
8. Eckert C., Lecerf M., Dubost L., Arthur M. and Mesnage, S. (2006) Functional analysis of AtlA, the major *N*-acetylglucosaminidase of *Enterococcus faecalis*. *J Bacteriol*. **188**(24):8513-8519
