# Supplementary material for: Enterococcal cell wall remodelling underpins pathogenesis via the release of the Enteroccocal Polysaccharide Antigen (EPA)
Source: PLoS Pathog. 2025 Jun 23;21(6):e1012771. doi: 10.1371/journal.ppat.1012771 (PMC12208459; doi:10.1371/journal.ppat.1012771)
Supplement: S1 File — Zipped raw data used to make figures 1–10 are provided in individual folders for each figure. (ZIP) [file ppat.1012771.s013.zip › Data Smith et al/Fig. 7/Western Blots.pptx]

## Slide 1
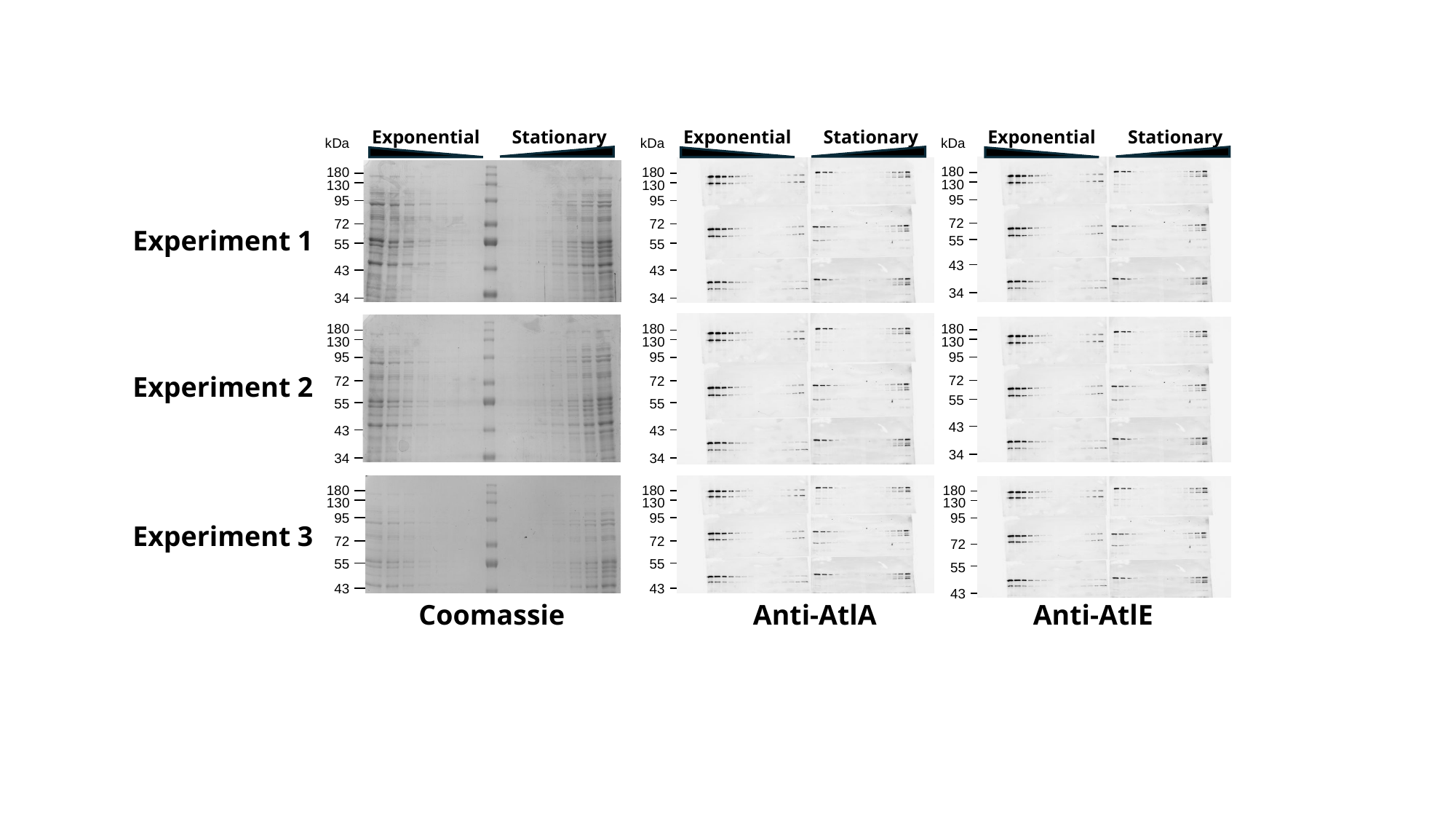

Exponential
Stationary
Exponential
Stationary
Exponential
Stationary
kDa
kDa
kDa
180
180
180
130
130
130
95
95
95
72
72
72
Experiment 1
55
55
55
43
43
43
34
34
34
180
180
180
130
130
130
95
95
95
Experiment 2
72
72
72
55
55
55
43
43
43
34
34
34
180
180
180
130
130
130
95
95
95
Experiment 3
72
72
72
55
55
55
43
43
43
Coomassie
Anti-AtlA
Anti-AtlE
