# Supplementary material for: Enterococcal cell wall remodelling underpins pathogenesis via the release of the Enteroccocal Polysaccharide Antigen (EPA)
Source: PLoS Pathog. 2025 Jun 23;21(6):e1012771. doi: 10.1371/journal.ppat.1012771 (PMC12208459; doi:10.1371/journal.ppat.1012771)
Supplement: Attachment — Submitted filename: PlosPath_EPA.docx [file ppat.1012771.s018.docx]

Summary

Overall this is a well written manuscript that characterizes a new peptidoglycan hydrolase named AtlE that is conserved in Enterococci. It shows that AtlE affects cell chaining during stationary phase when the major AtlA is inactive, and that it displays N-acetylmuramidase activity. Cell wall degradation by this enzyme is strain specific and dependent on EPA decorations. When tested in a zebrafish systemic infection model, deletion of *atlE* leads to a decrease in lethality, and exogenous EPA-decorated cell wall fragments increase WT lethality.

Major

1. The authors show that AtlE is important for control of cell chaining in stationary phase in the absence of AtlA. Can we consider AtlA and AtlE functionally redundant during stationary phase, where AtlE functions as a secondary autolysin that kicks in when AtlA is inactive? Is the LC-MS digestion profile of AtlE similar to cell wall digestions with AtlA? Drawing from what is known from studies with AtlB and AtlC, is there a possible physiologic explanation that would explain why the cell might require two autolysins during stationary phase?
2. The authors hypothesize that AtlE releases EPA to the environment and this in turn increases lethality of *E. faecalis*. While it is clear that AtlE and EPA-decorated cell wall fragments play a prominent role during systemic infection in the zebrafish model, the link between the two factors during *in vivo* infection has not been directly demonstrated. Have you tested if co-injection of WT EPA-decorated cell wall fragments restore lethality of the ∆*atlE* strain? And conversely, if AtlA and AtlE happen to be functionally redundant, do EPA-decorated cell wall fragments of the ∆*atlE* strain (released by AtlA) boost virulence of the WT strain?
3. At multiple points in the manuscript, the authors claim that the released cell wall fragments act as decoy molecules to inhibit the immune response, however no data are provided to substantiate this interpretation. Experiments to investigate should be performed. For example, previous work in this group has advanced our knowledge on the role of EPA during infection and interaction with macrophages. Do the EPA-decorated cell wall fragments inhibit the host immune system as opposed to the cell wall-attached EPA polymer. Do released EPA fragments protect from phagocytosis and charged antimicrobial peptides? This might help explain why the WT strain that already produces EPA-decorated cell wall fragments is more virulent when exogenous fragments are co-injected. Is it a matter of concentration?

Minor

1. Since AtlE requires EPA decorations to hydrolyse peptidoglycan and AtlE is active during stationary phase, do you know if EPA surface exposure or EPA decorations are more abundant during stationary phase than during exponential phase?
2. Why was it necessary to use the cell wall substrate of a weakened cell wall strain for the zymogram assay in Fig. 1? Similarly, the OG1RF AtlE was able to digest the weakened cell wall of the JH2-2 strain in Figure S2, even though AtlE was later shown to be strain specific. Is this cross-reactivity due to the weak cell wall of this PBP mutant strain?
3. Fig. 3: why t test instead of ANOVA?
4. L139: typo “altA and altE”
5. L149: typo “led had”
6. L306: typo “he injection”
