# Supplementary material for: Enterococcal cell wall remodelling underpins pathogenesis via the release of the Enteroccocal Polysaccharide Antigen (EPA)
Source: PLoS Pathog. 2025 Jun 23;21(6):e1012771. doi: 10.1371/journal.ppat.1012771 (PMC12208459; doi:10.1371/journal.ppat.1012771)
Supplement: Attachment — Submitted filename: Response to reviewers.docx [file ppat.1012771.s020.docx]

*Please note that line numbers refer to the revised manuscript with track changes*

**Part I - Summary**

In this study, the authors investigated the function of AtlE peptidoglycan hydrolase in *Enterococcus faecalis*. The experiments showed that AtlE acts together with AtlA to promote digestion of enterococcal chains at the stationary growth stage. The authors also demonstrated that AtlE is muramidase which is important for virulence in the zebrafish model of infection. The report is important in that the function of peptidoglycan hydrolases is generally under-investigated in enterococci.

However, the study **did not prove that AtlE is directly involved in the release of cell wall fragments**.

We have now provided direct evidence that EPA is released in culture supernatants and that this process is abolished in the *atlE* mutant (Figure 8).

It is also unclear if virulence in zebrafish models depends on **EPA or peptidoglycan fragments**.

A previous study showed that soluble peptidoglycan fragments have no impact on bacterial virulence in the zebrafish model of infection (Boldock *et al*., 2018 ***Nature Microbiol***; doi: 10.1038/s41564-018-0198-3, see Figure S1). Our results agree with this conclusion since *epaR* cell wall fragments (which contain peptidoglycan fragments and EPA devoid of decorations) have no effect on zebrafish lethality, whether injected on their own or with WT cells. A slight difference is found between the WT and *epaR* muropeptide profiles (a slight increase of crosslinked material, see below) but if anything, this would be expected to increase the virulence of the *epaR* mutant. Which is clearly not the case.

Our comparative analysis involving WT and *epaR* cell wall fragments strongly supports the idea that virulence depends on EPA, not peptidoglycan.

A202nm (mAU)

| Time (min) | WT A | epaR A |
| --- | --- | --- |
| 0.008267 | 0 | 1500 |
| 0.0166 | -0.0499209 | 1499.95 |
| 0.024933 | -0.0942311 | 1499.9 |
| 0.033267 | -0.130011 | 1499.86 |
| 0.0416 | -0.160498 | 1499.83 |
| 0.049933 | -0.187467 | 1499.8 |
| 0.058267 | -0.217692 | 1499.76 |
| 0.0666 | -0.252299 | 1499.73 |
| 0.074933 | -0.287858 | 1499.69 |
| 0.083267 | -0.324899 | 1499.64 |
| 0.0916 | -0.365034 | 1499.6 |
| 0.099933 | -0.406999 | 1499.56 |
| 0.108267 | -0.448176 | 1499.51 |
| 0.1166 | -0.488343 | 1499.46 |
| 0.124933 | -0.528259 | 1499.42 |
| 0.133267 | -0.569416 | 1499.38 |
| 0.1416 | -0.612856 | 1499.33 |
| 0.149933 | -0.650884 | 1499.29 |
| 0.158267 | -0.688705 | 1499.25 |
| 0.1666 | -0.727974 | 1499.2 |
| 0.174933 | -0.767419 | 1499.17 |
| 0.183267 | -0.805213 | 1499.12 |
| 0.1916 | -0.842784 | 1499.08 |
| 0.199933 | -0.878518 | 1499.04 |
| 0.208267 | -0.918133 | 1499 |
| 0.2166 | -0.953353 | 1498.96 |
| 0.224933 | -0.988504 | 1498.92 |
| 0.233267 | -1.02498 | 1498.89 |
| 0.2416 | -1.05958 | 1498.85 |
| 0.249933 | -1.09305 | 1498.81 |
| 0.258267 | -1.12719 | 1498.78 |
| 0.2666 | -1.15987 | 1498.74 |
| 0.274933 | -1.19355 | 1498.7 |
| 0.283267 | -1.22627 | 1498.67 |
| 0.2916 | -1.25775 | 1498.63 |
| 0.299933 | -1.2901 | 1498.6 |
| 0.308267 | -1.32087 | 1498.57 |
| 0.3166 | -1.34754 | 1498.54 |
| 0.324933 | -1.37684 | 1498.51 |
| 0.333267 | -1.40308 | 1498.47 |
| 0.3416 | -1.43744 | 1498.44 |
| 0.349933 | -1.46967 | 1498.4 |
| 0.358267 | -1.50275 | 1498.36 |
| 0.3666 | -1.53969 | 1498.33 |
| 0.374933 | -1.57102 | 1498.29 |
| 0.383267 | -1.60484 | 1498.26 |
| 0.3916 | -1.63633 | 1498.23 |
| 0.399933 | -1.66306 | 1498.2 |
| 0.408267 | -1.69086 | 1498.17 |
| 0.4166 | -1.71709 | 1498.14 |
| 0.424933 | -1.74449 | 1498.11 |
| 0.433267 | -1.77259 | 1498.09 |
| 0.4416 | -1.79684 | 1498.06 |
| 0.449933 | -1.81995 | 1498.03 |
| 0.458267 | -1.84446 | 1498.01 |
| 0.4666 | -1.86861 | 1497.98 |
| 0.474933 | -1.89094 | 1497.96 |
| 0.483267 | -1.91376 | 1497.93 |
| 0.4916 | -1.93896 | 1497.91 |
| 0.499933 | -1.95993 | 1497.88 |
| 0.508267 | -1.98128 | 1497.86 |
| 0.5166 | -2.0039 | 1497.83 |
| 0.524933 | -2.02876 | 1497.81 |
| 0.533267 | -2.05086 | 1497.78 |
| 0.5416 | -2.0701 | 1497.76 |
| 0.549933 | -2.0966 | 1497.74 |
| 0.558267 | -2.11743 | 1497.71 |
| 0.5666 | -2.14158 | 1497.69 |
| 0.574933 | -2.164 | 1497.66 |
| 0.583267 | -2.18573 | 1497.63 |
| 0.5916 | -2.21548 | 1497.59 |
| 0.599933 | -2.24655 | 1497.55 |
| 0.608267 | -2.2866 | 1497.5 |
| 0.6166 | -2.33273 | 1497.43 |
| 0.624933 | -2.3905 | 1497.36 |
| 0.633267 | -2.45536 | 1497.28 |
| 0.6416 | -2.5301 | 1497.19 |
| 0.649933 | -2.60927 | 1497.09 |
| 0.658267 | -2.68981 | 1497 |
| 0.6666 | -2.7743 | 1496.91 |
| 0.674933 | -2.85402 | 1496.82 |
| 0.683267 | -2.93635 | 1496.73 |
| 0.6916 | -3.0101 | 1496.66 |
| 0.699933 | -3.07963 | 1496.58 |
| 0.708267 | -3.14631 | 1496.52 |
| 0.7166 | -3.20144 | 1496.46 |
| 0.724933 | -3.25499 | 1496.41 |
| 0.733267 | -3.30188 | 1496.36 |
| 0.7416 | -3.34194 | 1496.32 |
| 0.749933 | -3.37801 | 1496.29 |
| 0.758267 | -3.40802 | 1496.25 |
| 0.7666 | -3.43699 | 1496.23 |
| 0.774933 | -3.45982 | 1496.21 |
| 0.783267 | -3.48211 | 1496.19 |
| 0.7916 | -3.49599 | 1496.17 |
| 0.799933 | -3.51357 | 1496.15 |
| 0.808267 | -3.52443 | 1496.14 |
| 0.8166 | -3.53362 | 1496.13 |
| 0.824933 | -3.53852 | 1496.13 |
| 0.833267 | -3.53977 | 1496.12 |
| 0.8416 | -3.54073 | 1496.11 |
| 0.849933 | -3.53549 | 1496.11 |
| 0.858267 | -3.52258 | 1496.1 |
| 0.8666 | -3.49957 | 1496.1 |
| 0.874933 | -3.47109 | 1496.09 |
| 0.883267 | -3.42751 | 1496.09 |
| 0.8916 | -3.37493 | 1496.09 |
| 0.899933 | -3.31511 | 1496.09 |
| 0.908267 | -3.24455 | 1496.1 |
| 0.9166 | -3.16856 | 1496.1 |
| 0.924933 | -3.08771 | 1496.11 |
| 0.933267 | -3.00733 | 1496.12 |
| 0.9416 | -2.92506 | 1496.13 |
| 0.949933 | -2.84645 | 1496.15 |
| 0.958267 | -2.78242 | 1496.16 |
| 0.9666 | -2.72652 | 1496.17 |
| 0.974933 | -2.68537 | 1496.19 |
| 0.983267 | -2.66231 | 1496.2 |
| 0.9916 | -2.65071 | 1496.21 |
| 0.999933 | -2.64762 | 1496.21 |
| 1.00827 | -2.66038 | 1496.21 |
| 1.0166 | -2.68343 | 1496.2 |
| 1.02493 | -2.71333 | 1496.2 |
| 1.03327 | -2.7495 | 1496.19 |
| 1.0416 | -2.78697 | 1496.19 |
| 1.04993 | -2.82805 | 1496.18 |
| 1.05827 | -2.87095 | 1496.17 |
| 1.0666 | -2.90989 | 1496.16 |
| 1.07493 | -2.94987 | 1496.16 |
| 1.08327 | -2.98512 | 1496.15 |
| 1.0916 | -3.01902 | 1496.14 |
| 1.09993 | -3.0504 | 1496.14 |
| 1.10827 | -3.0772 | 1496.13 |
| 1.1166 | -3.1012 | 1496.13 |
| 1.12493 | -3.12598 | 1496.13 |
| 1.13327 | -3.1447 | 1496.12 |
| 1.1416 | -3.15862 | 1496.12 |
| 1.14993 | -3.17171 | 1496.12 |
| 1.15827 | -3.17959 | 1496.12 |
| 1.1666 | -3.18372 | 1496.12 |
| 1.17493 | -3.18161 | 1496.13 |
| 1.18327 | -3.16625 | 1496.15 |
| 1.1916 | -3.13909 | 1496.18 |
| 1.19993 | -3.09505 | 1496.23 |
| 1.20827 | -3.01077 | 1496.3 |
| 1.2166 | -2.80237 | 1496.42 |
| 1.22493 | -2.17997 | 1496.71 |
| 1.23327 | -0.179912 | 1497.81 |
| 1.2416 | 5.44735 | 1501.76 |
| 1.24993 | 17.2825 | 1511.75 |
| 1.25827 | 35.8036 | 1529.17 |
| 1.2666 | 58.7358 | 1552.08 |
| 1.27493 | 82.8305 | 1576.89 |
| 1.28327 | 105.769 | 1600.76 |
| 1.2916 | 126.392 | 1622.11 |
| 1.29993 | 144.266 | 1640.27 |
| 1.30827 | 159.427 | 1655.27 |
| 1.3166 | 171.855 | 1667.21 |
| 1.32493 | 181.516 | 1676.07 |
| 1.33327 | 188.423 | 1681.69 |
| 1.3416 | 192.681 | 1683.86 |
| 1.34993 | 194.399 | 1682.47 |
| 1.35827 | 193.641 | 1677.75 |
| 1.3666 | 190.391 | 1671.19 |
| 1.37493 | 184.782 | 1666.01 |
| 1.38327 | 177.217 | 1664.95 |
| 1.3916 | 168.357 | 1669.86 |
| 1.39993 | 158.945 | 1679.12 |
| 1.40827 | 149.618 | 1689.71 |
| 1.4166 | 140.887 | 1698.96 |
| 1.42493 | 133.07 | 1705.27 |
| 1.43327 | 126.331 | 1708 |
| 1.4416 | 120.684 | 1707.97 |
| 1.44993 | 116.073 | 1707.15 |
| 1.45827 | 112.496 | 1707.16 |
| 1.4666 | 110.033 | 1709.1 |
| 1.47493 | 108.808 | 1713.64 |
| 1.48327 | 109.048 | 1720.3 |
| 1.4916 | 111.133 | 1727.36 |
| 1.49993 | 115.284 | 1732.84 |
| 1.50827 | 121.137 | 1735.38 |
| 1.5166 | 127.834 | 1734.61 |
| 1.52493 | 134.31 | 1730.92 |
| 1.53327 | 139.648 | 1725.03 |
| 1.5416 | 143.287 | 1717.61 |
| 1.54993 | 145.059 | 1709.11 |
| 1.55827 | 144.914 | 1699.84 |
| 1.5666 | 142.702 | 1690.1 |
| 1.57493 | 138.407 | 1680.2 |
| 1.58327 | 132.378 | 1670.44 |
| 1.5916 | 125.227 | 1660.97 |
| 1.59993 | 117.57 | 1651.88 |
| 1.60827 | 109.887 | 1643.13 |
| 1.6166 | 102.511 | 1634.71 |
| 1.62493 | 95.6597 | 1626.59 |
| 1.63327 | 89.4179 | 1618.76 |
| 1.6416 | 83.7731 | 1611.27 |
| 1.64993 | 78.7589 | 1604.23 |
| 1.65827 | 74.446 | 1597.75 |
| 1.6666 | 70.8932 | 1591.95 |

**Rp-HPLC muropeptide profiles of the OG1RF (WT) and *epaR* mutant grown in exponential phase**

Related to the comments above, I don’t think the title is fully appropriate.

Based on the new experimental evidence provided (the release of EPA in culture supernatants) and the evidence that AtlE cleaves peptidoglycan during growth, we now feel that the title is fully appropriate. We would, however, be happy to consider suggestions from the reviewer for an alternative title.

Finally, there is no explanation **if activity of AtlE in the release of cell wall fragments requires AtlA or other enzymes** expressed in the stationary growth phase.

Whether AtlE requires AtlA or (an)other peptidoglycan hydrolases to release cell wall fragments is an interesting question. However, investigating a potential contribution of AtlA or other hydrolases would not change any of the conclusions drawn in this study. We have now shown that AtlA and AtlE are differentially expressed during growth and display distinctive activities towards cell walls from exponential and stationary phase. We therefore feel that the revised version now provides some information about the specific roles of both enzymes. Documenting their overlapping roles is likely to make this manuscript more confusing.

**Part II – Major Issues: Key Experiments Required for Acceptance**

1. Line 308. I disagree that the experiment in Fig 7 shows that the increase of embryo lethality is mediated by soluble cell wall fragments required EPA decoration.

**Firstly,** this experiment should provide evidence that WT, the Δ*atlE* mutant and the complemented strain display differences in cell wall fragments.

We assume that the reviewer meant “that WT, the Δ*atlE* mutant and the complemented strain display differences in THEIR CAPACITY TO RELEASE cell wall fragments”?

This evidence is now shown in Figure 8. We did not include the complemented strain because the complementation requires the addition of anhydrotetracycline, which leads to the overexpression of AtlE.

**Secondly**, it is unclear how WT or Δ*epaR* mutant cell wall fragments were obtained for this assay. Please, provide also details how the concentration was determined and why different concentrations of WT or Δ*epaR* were used.

This is now described in detail in the materials and methods section (L. 691-701). Soluble cell wall fragments were freeze-dried and weighted. We generated a decent amount of cell walls (3.5mg for WT and 3.7mg for the Δ*epaR* cell walls), which makes us confident that the quantification was accurate.

**Thirdly**, if cell wall fragments contain EPA attached to peptidoglycan, the effect of both components (EPA and peptidoglycan) should be studied in this virulence models.

See comment above

**Lastly**, the study with the soluble fragments should be conducted with all groups (OG1RF, WT, Δ*epaR*) in a single experiment. Thus, I believe that the statement in Abstract that soluble cell wall fragments containing EPA decorations increase the virulence of *Enterococcus faecalis* has not proved yet.

The zebrafish experiments are conducted with at least 25 embryos per group and unfortunately the experiment suggested by the reviewer is not really feasible since it would require 5 sets of injections. Injecting more than 3 groups of embryos is relatively challenging and also implies that the last injections take place 1-2h after the first ones. This is why we did the experiments as shown in Figure 10. Given the high reproducibility of the zebrafish experiments (we systematically do 3 independent experiments), we are confident that the data shown is robust.

We have now provided evidence that the cell wall fragments from the WT strain can complement the Δ*atlE* mutation (Fig. 10B).

2. How deletion of AtlA and both enzymes, AtlA and AtlE, affect *E. faecalis* virulence in the zebrafish model? Do the enzymes work together to release cell wall fragments?

We showed that the formation of cell chains associated with the deletion of *atlA* leads to an increased uptake by phagocytes and a reduced virulence (Salamaga *et al*., PLoS Path, 2017, doi: 10.1371/journal.ppat.1006526; Figure 7). We therefore expect that the combination of both mutations in *atlA* and *atlE* will have synergistic effects. Again, we feel that this question is interesting but irrespective of the result, the conclusions based on the experimental data shown will remain unchanged.

3. Could authors compare the activities of AtlA and AtlE in exponential and stationary growth stages?

Extract cell walls in expo and stat, compare PG (proxy for activity?), test activity of purified enzymes?

We have carried out experiments to quantify the abundance of AtlA and AtlE during exponential and stationary phase and measure their enzymatic activities against cell walls extracted from these different growth phases. The data are presented in Figure 7. The muropeptide profiles of cells in exponential and stationary phase is virtually identical and shown below.

A202nm (mAU)

| Time (min) | Expo 1 | 24h 1 |
| --- | --- | --- |
| 0.008267 | 0 | 1470.88 |
| 0.0166 | -0.108985 | 1470.8 |
| 0.024933 | -0.203511 | 1470.74 |
| 0.033267 | -0.276299 | 1470.68 |
| 0.0416 | -0.340544 | 1470.62 |
| 0.049933 | -0.397926 | 1470.57 |
| 0.058267 | -0.457271 | 1470.52 |
| 0.0666 | -0.525692 | 1470.46 |
| 0.074933 | -0.601584 | 1470.4 |
| 0.083267 | -0.689387 | 1470.34 |
| 0.0916 | -0.770602 | 1470.27 |
| 0.099933 | -0.864532 | 1470.2 |
| 0.108267 | -0.957345 | 1470.14 |
| 0.1166 | -1.04411 | 1470.08 |
| 0.124933 | -1.13149 | 1470.01 |
| 0.133267 | -1.22413 | 1469.95 |
| 0.1416 | -1.30697 | 1469.89 |
| 0.149933 | -1.40368 | 1469.82 |
| 0.158267 | -1.48732 | 1469.77 |
| 0.1666 | -1.56488 | 1469.7 |
| 0.174933 | -1.65021 | 1469.64 |
| 0.183267 | -1.74091 | 1469.58 |
| 0.1916 | -1.81897 | 1469.52 |
| 0.199933 | -1.88968 | 1469.46 |
| 0.208267 | -1.97495 | 1469.41 |
| 0.2166 | -2.05325 | 1469.35 |
| 0.224933 | -2.1317 | 1469.29 |
| 0.233267 | -2.20726 | 1469.23 |
| 0.2416 | -2.29361 | 1469.19 |
| 0.249933 | -2.35994 | 1469.14 |
| 0.258267 | -2.42879 | 1469.08 |
| 0.2666 | -2.49886 | 1469.03 |
| 0.274933 | -2.57541 | 1468.98 |
| 0.283267 | -2.64717 | 1468.93 |
| 0.2916 | -2.72732 | 1468.88 |
| 0.299933 | -2.78703 | 1468.82 |
| 0.308267 | -2.8594 | 1468.78 |
| 0.3166 | -2.9183 | 1468.73 |
| 0.324933 | -2.97302 | 1468.69 |
| 0.333267 | -3.04067 | 1468.63 |
| 0.3416 | -3.09714 | 1468.57 |
| 0.349933 | -3.16812 | 1468.52 |
| 0.358267 | -3.23701 | 1468.46 |
| 0.3666 | -3.31144 | 1468.4 |
| 0.374933 | -3.38808 | 1468.34 |
| 0.383267 | -3.45869 | 1468.29 |
| 0.3916 | -3.52801 | 1468.24 |
| 0.399933 | -3.58806 | 1468.19 |
| 0.408267 | -3.64369 | 1468.15 |
| 0.4166 | -3.70285 | 1468.11 |
| 0.424933 | -3.7689 | 1468.07 |
| 0.433267 | -3.81844 | 1468.03 |
| 0.4416 | -3.87731 | 1468 |
| 0.449933 | -3.93528 | 1467.96 |
| 0.458267 | -3.98337 | 1467.92 |
| 0.4666 | -4.0339 | 1467.89 |
| 0.474933 | -4.08855 | 1467.85 |
| 0.483267 | -4.13691 | 1467.82 |
| 0.4916 | -4.18696 | 1467.79 |
| 0.499933 | -4.23137 | 1467.76 |
| 0.508267 | -4.27574 | 1467.73 |
| 0.5166 | -4.32819 | 1467.7 |
| 0.524933 | -4.38771 | 1467.67 |
| 0.533267 | -4.43399 | 1467.63 |
| 0.5416 | -4.47754 | 1467.6 |
| 0.549933 | -4.533 | 1467.57 |
| 0.558267 | -4.58181 | 1467.53 |
| 0.5666 | -4.63477 | 1467.5 |
| 0.574933 | -4.68206 | 1467.47 |
| 0.583267 | -4.73388 | 1467.44 |
| 0.5916 | -4.786 | 1467.39 |
| 0.599933 | -4.84591 | 1467.35 |
| 0.608267 | -4.91596 | 1467.28 |
| 0.6166 | -5.00566 | 1467.2 |
| 0.624933 | -5.11502 | 1467.11 |
| 0.633267 | -5.2425 | 1467 |
| 0.6416 | -5.38403 | 1466.88 |
| 0.649933 | -5.54456 | 1466.76 |
| 0.658267 | -5.71392 | 1466.63 |
| 0.6666 | -5.89672 | 1466.5 |
| 0.674933 | -6.07754 | 1466.37 |
| 0.683267 | -6.25428 | 1466.26 |
| 0.6916 | -6.42222 | 1466.15 |
| 0.699933 | -6.59142 | 1466.04 |
| 0.708267 | -6.73145 | 1465.94 |
| 0.7166 | -6.86196 | 1465.86 |
| 0.724933 | -6.9892 | 1465.78 |
| 0.733267 | -7.10431 | 1465.72 |
| 0.7416 | -7.19454 | 1465.66 |
| 0.749933 | -7.27628 | 1465.61 |
| 0.758267 | -7.35415 | 1465.57 |
| 0.7666 | -7.42881 | 1465.53 |
| 0.774933 | -7.48648 | 1465.5 |
| 0.783267 | -7.52642 | 1465.47 |
| 0.7916 | -7.57334 | 1465.45 |
| 0.799933 | -7.6134 | 1465.43 |
| 0.808267 | -7.64541 | 1465.42 |
| 0.8166 | -7.67623 | 1465.41 |
| 0.824933 | -7.69397 | 1465.4 |
| 0.833267 | -7.71412 | 1465.39 |
| 0.8416 | -7.72721 | 1465.4 |
| 0.849933 | -7.73221 | 1465.41 |
| 0.858267 | -7.74264 | 1465.44 |
| 0.8666 | -7.72459 | 1465.48 |
| 0.874933 | -7.69665 | 1465.54 |
| 0.883267 | -7.66075 | 1465.62 |
| 0.8916 | -7.59083 | 1465.72 |
| 0.899933 | -7.51306 | 1465.81 |
| 0.908267 | -7.42276 | 1465.91 |
| 0.9166 | -7.3298 | 1466 |
| 0.924933 | -7.24341 | 1466.09 |
| 0.933267 | -7.15754 | 1466.17 |
| 0.9416 | -7.09178 | 1466.23 |
| 0.949933 | -7.03921 | 1466.27 |
| 0.958267 | -7.00658 | 1466.31 |
| 0.9666 | -6.98342 | 1466.32 |
| 0.974933 | -6.97031 | 1466.33 |
| 0.983267 | -6.95505 | 1466.33 |
| 0.9916 | -6.95401 | 1466.32 |
| 0.999933 | -6.95686 | 1466.31 |
| 1.00827 | -6.96125 | 1466.3 |
| 1.0166 | -6.95723 | 1466.29 |
| 1.02493 | -6.97201 | 1466.29 |
| 1.03327 | -6.97765 | 1466.28 |
| 1.0416 | -6.98625 | 1466.28 |
| 1.04993 | -7.00568 | 1466.27 |
| 1.05827 | -7.00676 | 1466.26 |
| 1.0666 | -7.01739 | 1466.26 |
| 1.07493 | -7.03158 | 1466.26 |
| 1.08327 | -7.02117 | 1466.26 |
| 1.0916 | -7.03201 | 1466.25 |
| 1.09993 | -7.03653 | 1466.25 |
| 1.10827 | -7.03582 | 1466.25 |
| 1.1166 | -7.03641 | 1466.25 |
| 1.12493 | -7.03323 | 1466.24 |
| 1.13327 | -7.01823 | 1466.23 |
| 1.1416 | -7.01322 | 1466.22 |
| 1.14993 | -7.00328 | 1466.21 |
| 1.15827 | -6.98701 | 1466.2 |
| 1.1666 | -6.9616 | 1466.19 |
| 1.17493 | -6.92978 | 1466.19 |
| 1.18327 | -6.88507 | 1466.19 |
| 1.1916 | -6.81596 | 1466.21 |
| 1.19993 | -6.72585 | 1466.26 |
| 1.20827 | -6.61023 | 1466.33 |
| 1.2166 | -6.44422 | 1466.42 |
| 1.22493 | -6.13073 | 1466.52 |
| 1.23327 | -4.4763 | 1466.63 |
| 1.2416 | 4.16365 | 1466.99 |
| 1.24993 | 30.543 | 1468.37 |
| 1.25827 | 80.3816 | 1471.98 |
| 1.2666 | 147.746 | 1478.79 |
| 1.27493 | 220.434 | 1488.97 |
| 1.28327 | 288.023 | 1502.2 |
| 1.2916 | 344.708 | 1518.11 |
| 1.29993 | 388.767 | 1536.13 |
| 1.30827 | 420.257 | 1555.51 |
| 1.3166 | 440.273 | 1575.24 |
| 1.32493 | 450.269 | 1593.88 |
| 1.33327 | 452.299 | 1610.02 |
| 1.3416 | 448.708 | 1622.68 |
| 1.34993 | 441.515 | 1631.27 |
| 1.35827 | 431.996 | 1635.47 |
| 1.3666 | 420.731 | 1635.46 |
| 1.37493 | 407.979 | 1631.9 |
| 1.38327 | 393.982 | 1625.9 |
| 1.3916 | 379.073 | 1618.67 |
| 1.39993 | 363.776 | 1611.44 |
| 1.40827 | 348.701 | 1605.07 |
| 1.4166 | 334.276 | 1600.04 |
| 1.42493 | 320.751 | 1596.6 |
| 1.43327 | 308.229 | 1595 |
| 1.4416 | 296.688 | 1595.54 |
| 1.44993 | 286.216 | 1598.1 |
| 1.45827 | 276.748 | 1602.34 |
| 1.4666 | 268.074 | 1608.01 |
| 1.47493 | 259.747 | 1614.9 |
| 1.48327 | 251.536 | 1622.55 |
| 1.4916 | 243.393 | 1630.24 |
| 1.49993 | 235.316 | 1637.17 |
| 1.50827 | 227.342 | 1642.63 |
| 1.5166 | 219.482 | 1646.2 |
| 1.52493 | 211.651 | 1647.7 |
| 1.53327 | 203.742 | 1647.23 |
| 1.5416 | 195.629 | 1644.98 |
| 1.54993 | 187.226 | 1641.18 |
| 1.55827 | 178.504 | 1636.02 |
| 1.5666 | 169.528 | 1629.69 |
| 1.57493 | 160.502 | 1622.46 |
| 1.58327 | 151.734 | 1614.69 |
| 1.5916 | 143.481 | 1606.72 |
| 1.59993 | 135.962 | 1598.86 |
| 1.60827 | 129.259 | 1591.34 |
| 1.6166 | 123.425 | 1584.33 |
| 1.62493 | 118.463 | 1577.94 |
| 1.63327 | 114.4 | 1572.2 |
| 1.6416 | 111.231 | 1567.09 |
| 1.64993 | 108.977 | 1562.58 |
| 1.65827 | 107.658 | 1558.72 |
| 1.6666 | 107.251 | 1555.58 |

**Rp-HPLC muropeptide profiles of the OG1RF (WT) cells grown in exponential and stationary phase.**

4. Supplementary Fig 2 demonstrates that OG1RF supernatant is active with Δ*ponA* Δ*pbpF* Δ*pbpZ* which was constructed in JH2-2 background. However, Fig 6B shows that AtlE is poorly active with JH2-2 cell wall material. Please explain this discrepancy.

Apologies for this mistake! To detect AtlE activity in JH2-2 AND OG1RF extracts, zymograms were always carried out with homologous cell walls. Fig. S2 has been amended to indicate that OG1RF cell walls were used, not the triple mutant. Fig. S2 clearly shows the presence/absence of AtlE activities when expected but the zymograms were technically challenging to perform and image, precisely because we had to use WT OG1RF cell walls.

**Part III – Minor Issues: Editorial and Data Presentation Modifications**

1. Fig.1 A. Please indicate the growth conditions used to collect culture supernatants for this assay.

Done. All strains were grown overnight and zymograms were incubated for 72h at 37°C.

2. Fig. 2. Please show the whole gel for 36h conditions of renaturation. Indicate the growth conditions used to collect culture supernatants for this assay.

Done. All strains were grown overnight.

3. Please indicate the conditions for zymogram experiments in Supplementary Fig.1 and 2 (a renaturation time and the growth conditions used to collect culture supernatants).

Done. All strains were grown overnight and zymograms were incubated for 72h at 37°C

Please explain whether

4. Fig.3. FSC histograms of data depicted in Fig. 3 should be provided in Supplementary material.

Raw data have been provided as supplementary material.

5. Fig. 3 A. Please indicate the concentration of ATc used to induce atlE expression.

As stated in the Materials and Methods, precultures were grown in the presence of 10 ng/mL of ATc and cultures for flow cytometry in 50 ng/mL. This has been added in the legend.

6. Fig. 3 B. Please show the Δ*atlA* Δ*atlE* mutant.

Figure 3B has been amended to show the non-complemented Δ*atlA* Δ*atlE* mutant.

7. Line 150. The authors conclude that the deletion of *atlE* alone was associated with a minor but significant increase in chain length in stationary phase. Please provide the analysis of the Δ*atlE* mutant complemented with *atlE* (statistics).

We have re-analyzed the data as suggested by the editor using a one-way ANOVA rather than *t*-tests. There is NO significant impact of *atlE* deletion in chain length during both exponential AND stationary phase. A significant effect of the *atlE* deletion is only seen when combined with the atlA mutation.

8. Line 306. Edit “he” typo.

Done.

9. Line 511. Zebrafish experiments. Please provide details of this experiment. The reference 20 is not correct. Indicate the growth conditions of bacterial strains used for this experiment. Describe how soluble cell wall fragments were obtained for zebrafish experiment and how the concentration of cell wall fragments was determined.

The correct reference has been added (18, not 20).

All the details to prepare and quantify cell walls are provided in the revised manuscript (page 24, L. 691-701)

10. Reference Supplemental Fig 5,6, 7 in the text.

Done (now S8, S9, S10, S11).

**Response to reviewer #3**

**Part I - Summary**

This is an interesting study by Smith et al that describes the discovery and activity of a new peptidoglycan hydrolase linked to surface polysaccharides in Enterococcus faecalis. This is relevant to E. faecalis pathogenesis as Epa is important for virulence and is linked to biofilm formation, antibiotic resistance, and phage resistance. In Ef, the peptidoglycan hydrolase AtlA is known to contribute to septum division and Ef chain length/morphology. A nice summary of AtlA activity is provided. The authors show that an atlABC knockout still forms diplococci/short chains in stationary phase, suggesting the presence of another peptidoglycan hydrolase active in this growth phase. The authors used zymography to identify peptidoglycan hydrolase activity from an atlABC deletion strain and performed mass spec to identify putative hydrolases. They confirm activity of a new hydrolase encoded in the variable region of the epa operon in E. faecalis and rename this gene product as AtlE. They show AtlE has a role in regulating chain length primarily in stationary phase, not exponential phase, although the effect is strongest when chain length is already misregulated in the absence of atlA (atlA atlE double mutant).

Interestingly, the authors show sequence and domain variability within the C-termini of AtlE variants from different strains and highlight strains OG1RF and JH2-2 as examples in this study (OG1RF encodes a DUF5776 domain whereas JH2-2 encodes a GW domain). The authors use recombinant AtlE from OG1RF to demonstrate that AtlE-OG1RF has N-acetylmuramidase activity on OG1RF cell walls, but not cell walls derived from JH2-2. The authors then show that AtlE-OG1RF has no activity against cell walls purified from a strain that produces modified Epa and posit that Epa decorations are required for AtlE activity. The authors then use NMR to show that AtlE does not affect surface display or structure of Epa in strain OG1RF. Finally, the authors demonstrate that AtlE affects virulence using an animal model. An *atlE* deletion model had attenuated virulence, and the authors propose that Epa-containing cell wall fragments released by AtlE may be important for virulence.

In general, this is a detailed manuscript highlighting a new peptidoglycan hydrolase that contributes to *E. faecalis* pathogenesis and is linked to the virulence factor Epa. The biochemical data supporting AtlE activity is clear. A minor weakness of the manuscript is the presentation of Figure 4, as the differences between AtlE-OG1RF and AtlE-JH2-2 are somewhat obscured.

We have changed the colors to make them colorblind friendly. The C-terminal domains (GW or DUF5776) are now indicated in the revised Figure (4B). The text has been modified to highlight the key conclusions of our sequence analyses (L. 212-219): (i) amino acid sequence similarity between GW or DUF5776 domains are sometimes low; (ii) irrespective of the sequence similarity, the structures of repeats within a C-terminal domain are virtually identical; (iii) GW and DUF5776 domains are structurally unrelated.

Given the differences in C-terminal domains between JH2-2 and OG1RF, and that AtlE-OG1RF did not have activity against cell wall fragments from JH2-2, it seems that the paper would benefit from an increased discussion of Epa and cell wall differences between these strains, and speculation of what the recognition motif is for AtlE.

A section of the discussion is dedicated to the role of EPA recognition by the C-terminal domain as an essential step for PG hydrolysis (L. 421-427). Whilst the structure of EPA in OG1RF has been recently elucidated (Davis et al., 2025, <https://doi.org/10.1016/j.carbpol.2024.122686>) no information is available for JH2-2. We therefore feel that any discussion about the binding motif recognised by AtlE would be highly speculative and potentially controversial. We have started to explore this question but the characterization of interactions between proteins and complex carbohydrates such as EPA are extremely challenging so we expect that it will take several years to elucidate EPA recognition by AtlE. A substantial amount of work will be required to explore the functional diversity of AtlE C-terminal binding domains.

**Part II – Major Issues: Key Experiments Required for Acceptance**

1) Based on data in Figure 7 and previously published work in reference 6, authors propose that cell wall fragments containing Epa are released into supernatants by AtlE, and that these fragments contribute to virulence. The authors show numerous zymograms demonstrating hydrolase activity in culture supernatants, but it seems important to quantify or otherwise show the presence of Epa or Epa-containing cell wall fragments in supernatants.

We have now provided evidence that the WT cells release EPA in their culture supernatants whilst EPA cannot be detected in supernatants from the Δ*atlE* mutant (Fig. 8).

**Part III – Minor Issues: Editorial and Data Presentation Modifications**

1) Given that *atlE* is encoded in the *epa* operon, would an *epa* name not be more appropriate given the nomenclature used for other genes in this region?

Previous studies have named genes in the locus encoding EPA decorations as *“epaXYZ*”, with letters reflecting the position of the genes considered. However, since this locus contains a variable number of genes depending on the strain considered, this system could become confusing since the letter would not always reflect the position of the *atlE* hydrolase in all *epa* loci. Renaming *atlE* as *epaU* would not make sense for several strains. Whilst this nomenclature is fine with conserved genes (encoding the rhamnose backbone), we feel that it makes more sense to use *atlE*, which also refers to the PG hydrolase activity of the enzyme (Atl is often used to name autolysin genes).

2) It would be helpful to show a comparison of the entire Epa operon in OG1RF and JH2-2 with the location of *atlE* annotated. This would allow readers to put into context where *atlE* is with respect to other Epa genes.

We have added a supplementary figure with a comparative description of the 2 loci (Fig. S2).

3) Figure 1: Viewing or printing the manuscript in grayscale will obscure the red text. Using brackets and text directly in panel B may prevent confusion for readers.

Figure 1 has been modified. Proteins in red are now bold, and they have been explicitly cited in the legend.

4) Figure 3: A and B labels are missing from the figure panels.

Figure 3 has been modified.

5) Figure 4: Similar to the above comment, the color convention between panels A, B, and C may be lost to colorblind readers or if the manuscript is viewed in grayscale. Directly labeling OG1RF and JH2-2 in panel B with the DUF5776 and GW would be beneficial given that R1-R4 are used to label repeat region in both diagrams.

We have modified panel A and used colorblind-friendly colors. Panel B has been labelled with DUF5776 and GW.

In panel C, it is difficult to tell that there are 2 structures shown in the overlays. Colors with higher contrast (such as using black for OG1RF or JH2-2) would be helpful.

The two structures cannot be distinguished, precisely because they are virtually identical! For this reason, we feel that colors don’t matter at all in panel C. We have added a comment to highlight the fact that the structures compared are virtually identical, with minor differences in loops corresponding to regions that remain difficult to predict with confidence by Alphafold.

6) Lines 229-230: more info (1-2 sentences) on the biochemical nature of Epa decorations would be helpful here.

We added a sentence (L. 266-268).As indicated in the text, the structure EPA produced by the mutant used is described in ref 20.

7) Figure S1: 0252 is listed twice in the legend (instead of 0114).

Figure S1 legend has been modified.
