# Supplementary material for: Enterococcal cell wall remodelling underpins pathogenesis via the release of the Enteroccocal Polysaccharide Antigen (EPA)
Source: PLoS Pathog. 2025 Jun 23;21(6):e1012771. doi: 10.1371/journal.ppat.1012771 (PMC12208459; doi:10.1371/journal.ppat.1012771)
Supplement: Attachment — Submitted filename: Response_to_Reviewers_auresp_2.docx [file ppat.1012771.s021.docx]

Response to Reviewer #1

**Part I – Summary**

In the revised version of the manuscript, Robert Smith et al. have provided additional data and clarifications that convincingly address the key concerns about the original submission and have substantially improved the quality of the manuscript.

**Part II – Major Issues: Key Experiments Required for Acceptance**

(No Response)

**Part III – Minor Issues: Editorial and Data Presentation Modifications**

1. Fig. 9 B and C. Scale bar is missing.

Fixed

2. Line 715. Indicate the method used to quantify cell wall fragments.

Done (cell walls were weighed once freeze-dried)

Response to Reviewer #2

**Part I – Summary**

Overall, this is a well written manuscript that characterizes a new peptidoglycan hydrolase named AtlE that is conserved in Enterococci. It shows that AtlE affects cell chaining during stationary phase when the major AtlA is inactive, and that it displays N-acetylmuramidase activity. Cell wall degradation by this enzyme is strain specific and dependent on EPA decorations. When tested in a zebrafish systemic infection model, deletion of *atlE* leads to a decrease in lethality, and exogenous EPA-decorated cell wall fragments increase WT lethality.

**Part II – Major Issues: Key Experiments Required for Acceptance**

The authors indirectly addressed most our previous comments, as many were quite similar to those of reviewer 1. However, it seems the authors did not receive/see our comments (which we uploaded as an attachment) since there is no point-by-point response to them.

We apologise for this! The .docx file attached to the decision email did not explicitly mention that it contained reviewer #2 comments from part II and we assumed these were comments from the editor. We included a point-by-point response that we submitted as a word file entitled “response to the editor” but the editorial team requested to include our response to the cover letter.

Our original point-by-point response sent with the revised manuscript is below.

Previous major comments listed here:

Major
1. The authors show that AtlE is important for control of cell chaining in stationary phase in the absence of AtlA. Can we consider AtlA and AtlE functionally redundant during stationary phase, where AtlE functions as a secondary autolysin that kicks in when AtlA is inactive?

There is most certainly an overlap between AtlA and AtlE activities, but AtlA and AtlE cannot be considered as functionally redundant given that single mutants have clear phenotypes. Both enzymes are produced during exponential and stationary phase and the balance between these two activities is expected to change during growth (based on our western blot, flow cytometry and in vitro assays). We have added a comment in the revised manuscript to discuss the partially redundant activity of both enzymes (L. 407-416).

Is the LC-MS digestion profile of AtlE similar to cell wall digestions with AtlA? Drawing from what is known from studies with AtlB and AtlC, is there a possible physiologic explanation that would explain why the cell might require two autolysins during stationary phase?

Our HPLC analyses indicate that there are no major differences between the WT, *atlA* and *atlE* mutants in exponential or stationary phase (see below) but the *in vitro* assays (Fig. 7) indicate that these 2 enzymes display distinct substrate preferences.

A202nm (mAU)

| Time (min) | Value (mAU) | Value (mAU) | Value (mAU) |
| --- | --- | --- | --- |
| 0.001633 | 5.0364 | 1003.43 | 2003.13 |
| 0.0033 | 5.04157 | 1003.45 | 2003.14 |
| 0.004967 | 5.0531 | 1003.45 | 2003.14 |
| 0.006633 | 5.05125 | 1003.46 | 2003.13 |
| 0.0083 | 5.04437 | 1003.47 | 2003.14 |
| 0.009967 | 5.02568 | 1003.47 | 2003.14 |
| 0.011633 | 5.05139 | 1003.47 | 2003.13 |
| 0.0133 | 5.05236 | 1003.43 | 2003.12 |
| 0.014967 | 5.05455 | 1003.46 | 2003.13 |
| 0.016633 | 5.0704 | 1003.46 | 2003.13 |
| 0.0183 | 5.06071 | 1003.44 | 2003.13 |
| 0.019967 | 5.06235 | 1003.42 | 2003.12 |
| 0.021633 | 5.07973 | 1003.41 | 2003.14 |
| 0.0233 | 5.06992 | 1003.42 | 2003.13 |
| 0.024967 | 5.07513 | 1003.43 | 2003.13 |
| 0.026633 | 5.09512 | 1003.43 | 2003.12 |
| 0.0283 | 5.09771 | 1003.44 | 2003.12 |
| 0.029967 | 5.10011 | 1003.45 | 2003.14 |
| 0.031633 | 5.09174 | 1003.45 | 2003.17 |
| 0.0333 | 5.09824 | 1003.45 | 2003.18 |
| 0.034967 | 5.11443 | 1003.46 | 2003.19 |
| 0.036633 | 5.11565 | 1003.46 | 2003.18 |
| 0.0383 | 5.09246 | 1003.47 | 2003.18 |
| 0.039967 | 5.12032 | 1003.46 | 2003.19 |
| 0.041633 | 5.14317 | 1003.47 | 2003.2 |
| 0.0433 | 5.14805 | 1003.48 | 2003.2 |
| 0.044967 | 5.15241 | 1003.5 | 2003.21 |
| 0.046633 | 5.12845 | 1003.51 | 2003.22 |
| 0.0483 | 5.12894 | 1003.52 | 2003.22 |
| 0.049967 | 5.15107 | 1003.52 | 2003.24 |
| 0.051633 | 5.17468 | 1003.51 | 2003.26 |
| 0.0533 | 5.17608 | 1003.53 | 2003.25 |
| 0.054967 | 5.18742 | 1003.53 | 2003.23 |
| 0.056633 | 5.17925 | 1003.55 | 2003.25 |
| 0.0583 | 5.16785 | 1003.58 | 2003.27 |
| 0.059967 | 5.1645 | 1003.58 | 2003.26 |
| 0.061633 | 5.17622 | 1003.56 | 2003.26 |
| 0.0633 | 5.20534 | 1003.57 | 2003.28 |
| 0.064967 | 5.2261 | 1003.59 | 2003.28 |
| 0.066633 | 5.2249 | 1003.62 | 2003.28 |
| 0.0683 | 5.24854 | 1003.62 | 2003.29 |
| 0.069967 | 5.26262 | 1003.62 | 2003.31 |
| 0.071633 | 5.25058 | 1003.62 | 2003.31 |
| 0.0733 | 5.26337 | 1003.63 | 2003.3 |
| 0.074967 | 5.25694 | 1003.64 | 2003.32 |
| 0.076633 | 5.26649 | 1003.64 | 2003.33 |
| 0.0783 | 5.2661 | 1003.66 | 2003.34 |
| 0.079967 | 5.26521 | 1003.65 | 2003.33 |
| 0.081633 | 5.29092 | 1003.63 | 2003.34 |
| 0.0833 | 5.27062 | 1003.66 | 2003.35 |
| 0.084967 | 5.28146 | 1003.66 | 2003.36 |
| 0.086633 | 5.26811 | 1003.67 | 2003.35 |
| 0.0883 | 5.27241 | 1003.67 | 2003.36 |
| 0.089967 | 5.27555 | 1003.68 | 2003.35 |
| 0.091633 | 5.29121 | 1003.68 | 2003.38 |
| 0.0933 | 5.31146 | 1003.67 | 2003.39 |
| 0.094967 | 5.30266 | 1003.68 | 2003.38 |
| 0.096633 | 5.31779 | 1003.67 | 2003.36 |
| 0.0983 | 5.3054 | 1003.68 | 2003.38 |
| 0.099967 | 5.31022 | 1003.7 | 2003.39 |
| 0.101633 | 5.33365 | 1003.69 | 2003.39 |
| 0.1033 | 5.34204 | 1003.69 | 2003.39 |
| 0.104967 | 5.3395 | 1003.71 | 2003.39 |
| 0.106633 | 5.34404 | 1003.72 | 2003.39 |
| 0.1083 | 5.3299 | 1003.69 | 2003.39 |
| 0.109967 | 5.35622 | 1003.72 | 2003.4 |
| 0.111633 | 5.35147 | 1003.72 | 2003.41 |
| 0.1133 | 5.34334 | 1003.74 | 2003.42 |
| 0.114967 | 5.32608 | 1003.75 | 2003.43 |
| 0.116633 | 5.3536 | 1003.75 | 2003.41 |
| 0.1183 | 5.3554 | 1003.73 | 2003.41 |
| 0.119967 | 5.38405 | 1003.71 | 2003.42 |
| 0.121633 | 5.39323 | 1003.73 | 2003.44 |
| 0.1233 | 5.39038 | 1003.74 | 2003.44 |
| 0.124967 | 5.38211 | 1003.71 | 2003.44 |
| 0.126633 | 5.38157 | 1003.72 | 2003.44 |
| 0.1283 | 5.38812 | 1003.73 | 2003.42 |
| 0.129967 | 5.40935 | 1003.74 | 2003.43 |
| 0.131633 | 5.41622 | 1003.74 | 2003.42 |
| 0.1333 | 5.40737 | 1003.74 | 2003.45 |
| 0.134967 | 5.42878 | 1003.79 | 2003.44 |
| 0.136633 | 5.40853 | 1003.78 | 2003.44 |
| 0.1383 | 5.42364 | 1003.77 | 2003.43 |
| 0.139967 | 5.4287 | 1003.77 | 2003.42 |
| 0.141633 | 5.42317 | 1003.79 | 2003.44 |
| 0.1433 | 5.43664 | 1003.78 | 2003.44 |
| 0.144967 | 5.41135 | 1003.78 | 2003.45 |
| 0.146633 | 5.40361 | 1003.76 | 2003.45 |
| 0.1483 | 5.42917 | 1003.76 | 2003.47 |
| 0.149967 | 5.42661 | 1003.77 | 2003.46 |
| 0.151633 | 5.44605 | 1003.78 | 2003.46 |
| 0.1533 | 5.4548 | 1003.81 | 2003.45 |
| 0.154967 | 5.48085 | 1003.8 | 2003.46 |
| 0.156633 | 5.4682 | 1003.8 | 2003.46 |
| 0.1583 | 5.45277 | 1003.81 | 2003.47 |
| 0.159967 | 5.47986 | 1003.82 | 2003.5 |
| 0.161633 | 5.48649 | 1003.82 | 2003.5 |
| 0.1633 | 5.49589 | 1003.82 | 2003.49 |
| 0.164967 | 5.50724 | 1003.81 | 2003.5 |
| 0.166633 | 5.49998 | 1003.82 | 2003.51 |
| 0.1683 | 5.50677 | 1003.8 | 2003.5 |
| 0.169967 | 5.48651 | 1003.81 | 2003.49 |
| 0.171633 | 5.49049 | 1003.79 | 2003.49 |
| 0.1733 | 5.49911 | 1003.79 | 2003.49 |
| 0.174967 | 5.51461 | 1003.81 | 2003.5 |
| 0.176633 | 5.5325 | 1003.82 | 2003.49 |
| 0.1783 | 5.50427 | 1003.84 | 2003.5 |
| 0.179967 | 5.51569 | 1003.82 | 2003.52 |
| 0.181633 | 5.50565 | 1003.83 | 2003.53 |
| 0.1833 | 5.51219 | 1003.83 | 2003.52 |
| 0.184967 | 5.52406 | 1003.83 | 2003.54 |
| 0.186633 | 5.52715 | 1003.85 | 2003.53 |
| 0.1883 | 5.55663 | 1003.83 | 2003.54 |
| 0.189967 | 5.54666 | 1003.85 | 2003.54 |
| 0.191633 | 5.55346 | 1003.84 | 2003.52 |
| 0.1933 | 5.55924 | 1003.83 | 2003.54 |
| 0.194967 | 5.55503 | 1003.85 | 2003.54 |
| 0.196633 | 5.53678 | 1003.87 | 2003.56 |
| 0.1983 | 5.55188 | 1003.87 | 2003.57 |
| 0.199967 | 5.56843 | 1003.84 | 2003.55 |
| 0.201633 | 5.57715 | 1003.82 | 2003.56 |
| 0.2033 | 5.59311 | 1003.83 | 2003.57 |
| 0.204967 | 5.59425 | 1003.86 | 2003.56 |
| 0.206633 | 5.5907 | 1003.88 | 2003.59 |
| 0.2083 | 5.6123 | 1003.88 | 2003.58 |
| 0.209967 | 5.6036 | 1003.87 | 2003.56 |
| 0.211633 | 5.58269 | 1003.88 | 2003.56 |
| 0.2133 | 5.61294 | 1003.89 | 2003.58 |
| 0.214967 | 5.60881 | 1003.89 | 2003.57 |
| 0.216633 | 5.61086 | 1003.89 | 2003.55 |
| 0.2183 | 5.60665 | 1003.87 | 2003.56 |
| 0.219967 | 5.62423 | 1003.88 | 2003.56 |
| 0.221633 | 5.64092 | 1003.88 | 2003.56 |
| 0.2233 | 5.64334 | 1003.91 | 2003.57 |
| 0.224967 | 5.66247 | 1003.91 | 2003.59 |
| 0.226633 | 5.66764 | 1003.91 | 2003.6 |
| 0.2283 | 5.6553 | 1003.92 | 2003.61 |
| 0.229967 | 5.66091 | 1003.92 | 2003.6 |
| 0.231633 | 5.64006 | 1003.91 | 2003.59 |
| 0.2333 | 5.65333 | 1003.93 | 2003.6 |
| 0.234967 | 5.66696 | 1003.93 | 2003.6 |
| 0.236633 | 5.6751 | 1003.94 | 2003.61 |
| 0.2383 | 5.64682 | 1003.94 | 2003.59 |
| 0.239967 | 5.67057 | 1003.95 | 2003.6 |
| 0.241633 | 5.6823 | 1003.93 | 2003.59 |
| 0.2433 | 5.66725 | 1003.96 | 2003.61 |
| 0.244967 | 5.66116 | 1003.95 | 2003.61 |
| 0.246633 | 5.67858 | 1003.96 | 2003.61 |
| 0.2483 | 5.68783 | 1003.96 | 2003.61 |
| 0.249967 | 5.6977 | 1003.97 | 2003.64 |
| 0.251633 | 5.70482 | 1003.97 | 2003.64 |
| 0.2533 | 5.66919 | 1003.96 | 2003.63 |
| 0.254967 | 5.70235 | 1003.96 | 2003.65 |
| 0.256633 | 5.70862 | 1003.99 | 2003.64 |
| 0.2583 | 5.70387 | 1003.99 | 2003.65 |
| 0.259967 | 5.6831 | 1003.99 | 2003.64 |
| 0.261633 | 5.73028 | 1003.98 | 2003.62 |
| 0.2633 | 5.72637 | 1003.99 | 2003.63 |
| 0.264967 | 5.7309 | 1003.98 | 2003.64 |
| 0.266633 | 5.75022 | 1003.98 | 2003.63 |
| 0.2683 | 5.74127 | 1004 | 2003.65 |
| 0.269967 | 5.77778 | 1004 | 2003.65 |
| 0.271633 | 5.75626 | 1004 | 2003.64 |
| 0.2733 | 5.77132 | 1004.01 | 2003.66 |
| 0.274967 | 5.7692 | 1003.98 | 2003.64 |
| 0.276633 | 5.77799 | 1004.02 | 2003.65 |
| 0.2783 | 5.7967 | 1004.02 | 2003.66 |
| 0.279967 | 5.79061 | 1004.02 | 2003.66 |
| 0.281633 | 5.78505 | 1003.99 | 2003.65 |
| 0.2833 | 5.82541 | 1004 | 2003.65 |
| 0.284967 | 5.79849 | 1004 | 2003.66 |
| 0.286633 | 5.79555 | 1004.02 | 2003.66 |
| 0.2883 | 5.79779 | 1004.04 | 2003.68 |
| 0.289967 | 5.81839 | 1004.03 | 2003.68 |
| 0.291633 | 5.79985 | 1004.02 | 2003.7 |
| 0.2933 | 5.81056 | 1004.04 | 2003.71 |
| 0.294967 | 5.80615 | 1004.03 | 2003.71 |
| 0.296633 | 5.81897 | 1004.04 | 2003.7 |
| 0.2983 | 5.80267 | 1004.05 | 2003.69 |
| 0.299967 | 5.83192 | 1004.08 | 2003.68 |
| 0.301633 | 5.85829 | 1004.1 | 2003.67 |
| 0.3033 | 5.83132 | 1004.07 | 2003.68 |
| 0.304967 | 5.85943 | 1004.06 | 2003.69 |
| 0.306633 | 5.83374 | 1004.06 | 2003.69 |
| 0.3083 | 5.8368 | 1004.06 | 2003.71 |
| 0.309967 | 5.83266 | 1004.08 | 2003.73 |
| 0.311633 | 5.8633 | 1004.07 | 2003.72 |
| 0.3133 | 5.86327 | 1004.08 | 2003.71 |
| 0.314967 | 5.86622 | 1004.08 | 2003.72 |
| 0.316633 | 5.8634 | 1004.09 | 2003.72 |
| 0.3183 | 5.86793 | 1004.08 | 2003.71 |
| 0.319967 | 5.88295 | 1004.11 | 2003.72 |
| 0.321633 | 5.8785 | 1004.12 | 2003.72 |
| 0.3233 | 5.86495 | 1004.12 | 2003.75 |
| 0.324967 | 5.89124 | 1004.08 | 2003.74 |
| 0.326633 | 5.89289 | 1004.11 | 2003.74 |
| 0.3283 | 5.89695 | 1004.1 | 2003.72 |
| 0.329967 | 5.89265 | 1004.08 | 2003.72 |
| 0.331633 | 5.89747 | 1004.09 | 2003.74 |
| 0.3333 | 5.8804 | 1004.1 | 2003.75 |

A202nm (mAU)

| Time (min) | Value (mAU) | Value (mAU) | Value (mAU) |
| --- | --- | --- | --- |
| 0.001633 | 1002.9 | 2002.61 | 4.65686 |
| 0.0033 | 1002.9 | 2002.64 | 4.67497 |
| 0.004967 | 1002.9 | 2002.65 | 4.69513 |
| 0.006633 | 1002.9 | 2002.66 | 4.71495 |
| 0.0083 | 1002.91 | 2002.63 | 4.7203 |
| 0.009967 | 1002.9 | 2002.64 | 4.73022 |
| 0.011633 | 1002.91 | 2002.65 | 4.71841 |
| 0.0133 | 1002.92 | 2002.64 | 4.71152 |
| 0.014967 | 1002.92 | 2002.64 | 4.7241 |
| 0.016633 | 1002.92 | 2002.63 | 4.71683 |
| 0.0183 | 1002.92 | 2002.63 | 4.6906 |
| 0.019967 | 1002.93 | 2002.63 | 4.72702 |
| 0.021633 | 1002.92 | 2002.64 | 4.73458 |
| 0.0233 | 1002.93 | 2002.65 | 4.75433 |
| 0.024967 | 1002.93 | 2002.64 | 4.72696 |
| 0.026633 | 1002.94 | 2002.64 | 4.73582 |
| 0.0283 | 1002.92 | 2002.65 | 4.74538 |
| 0.029967 | 1002.96 | 2002.67 | 4.73973 |
| 0.031633 | 1002.94 | 2002.69 | 4.75895 |
| 0.0333 | 1002.96 | 2002.69 | 4.74471 |
| 0.034967 | 1002.93 | 2002.68 | 4.75276 |
| 0.036633 | 1002.95 | 2002.67 | 4.7641 |
| 0.0383 | 1002.95 | 2002.69 | 4.75368 |
| 0.039967 | 1002.95 | 2002.7 | 4.75445 |
| 0.041633 | 1002.96 | 2002.7 | 4.75031 |
| 0.0433 | 1002.96 | 2002.7 | 4.76674 |
| 0.044967 | 1002.97 | 2002.71 | 4.77836 |
| 0.046633 | 1002.96 | 2002.72 | 4.80047 |
| 0.0483 | 1002.97 | 2002.71 | 4.77562 |
| 0.049967 | 1002.98 | 2002.72 | 4.76349 |
| 0.051633 | 1003 | 2002.73 | 4.78779 |
| 0.0533 | 1002.99 | 2002.75 | 4.79291 |
| 0.054967 | 1003.02 | 2002.76 | 4.8331 |
| 0.056633 | 1002.99 | 2002.76 | 4.82703 |
| 0.0583 | 1003.01 | 2002.76 | 4.82374 |
| 0.059967 | 1003 | 2002.77 | 4.80726 |
| 0.061633 | 1003.01 | 2002.79 | 4.82732 |
| 0.0633 | 1003 | 2002.78 | 4.83115 |
| 0.064967 | 1003.01 | 2002.8 | 4.84868 |
| 0.066633 | 1003.03 | 2002.82 | 4.82528 |
| 0.0683 | 1003.03 | 2002.8 | 4.84696 |
| 0.069967 | 1003.04 | 2002.8 | 4.86221 |
| 0.071633 | 1003.05 | 2002.8 | 4.85603 |
| 0.0733 | 1003.05 | 2002.81 | 4.84906 |
| 0.074967 | 1003.07 | 2002.81 | 4.87114 |
| 0.076633 | 1003.06 | 2002.82 | 4.87124 |
| 0.0783 | 1003.08 | 2002.82 | 4.87598 |
| 0.079967 | 1003.08 | 2002.84 | 4.88774 |
| 0.081633 | 1003.06 | 2002.84 | 4.89568 |
| 0.0833 | 1003.08 | 2002.84 | 4.88149 |
| 0.084967 | 1003.1 | 2002.83 | 4.87156 |
| 0.086633 | 1003.09 | 2002.83 | 4.86515 |
| 0.0883 | 1003.09 | 2002.83 | 4.87218 |
| 0.089967 | 1003.1 | 2002.85 | 4.89912 |
| 0.091633 | 1003.08 | 2002.85 | 4.9127 |
| 0.0933 | 1003.08 | 2002.86 | 4.89227 |
| 0.094967 | 1003.1 | 2002.87 | 4.89784 |
| 0.096633 | 1003.11 | 2002.87 | 4.90595 |
| 0.0983 | 1003.11 | 2002.88 | 4.90503 |
| 0.099967 | 1003.12 | 2002.86 | 4.90227 |
| 0.101633 | 1003.13 | 2002.87 | 4.89664 |
| 0.1033 | 1003.13 | 2002.86 | 4.9204 |
| 0.104967 | 1003.11 | 2002.86 | 4.89911 |
| 0.106633 | 1003.12 | 2002.87 | 4.92558 |
| 0.1083 | 1003.13 | 2002.87 | 4.94055 |
| 0.109967 | 1003.14 | 2002.89 | 4.93067 |
| 0.111633 | 1003.15 | 2002.89 | 4.95374 |
| 0.1133 | 1003.14 | 2002.91 | 4.9612 |
| 0.114967 | 1003.14 | 2002.91 | 4.96576 |
| 0.116633 | 1003.13 | 2002.92 | 4.95445 |
| 0.1183 | 1003.12 | 2002.9 | 4.94625 |
| 0.119967 | 1003.13 | 2002.89 | 4.94944 |
| 0.121633 | 1003.15 | 2002.89 | 4.96791 |
| 0.1233 | 1003.12 | 2002.89 | 4.9629 |
| 0.124967 | 1003.13 | 2002.89 | 4.9646 |
| 0.126633 | 1003.13 | 2002.89 | 4.96095 |
| 0.1283 | 1003.13 | 2002.9 | 4.97608 |
| 0.129967 | 1003.15 | 2002.9 | 4.97216 |
| 0.131633 | 1003.14 | 2002.92 | 4.96805 |
| 0.1333 | 1003.17 | 2002.92 | 4.95094 |
| 0.134967 | 1003.16 | 2002.92 | 4.95338 |
| 0.136633 | 1003.17 | 2002.92 | 4.96103 |
| 0.1383 | 1003.16 | 2002.93 | 4.95871 |
| 0.139967 | 1003.16 | 2002.93 | 4.95141 |
| 0.141633 | 1003.17 | 2002.94 | 4.97091 |
| 0.1433 | 1003.18 | 2002.93 | 4.98899 |
| 0.144967 | 1003.16 | 2002.94 | 4.98106 |
| 0.146633 | 1003.16 | 2002.94 | 4.97415 |
| 0.1483 | 1003.19 | 2002.95 | 4.99794 |
| 0.149967 | 1003.2 | 2002.95 | 5.01738 |
| 0.151633 | 1003.19 | 2002.94 | 5.01184 |
| 0.1533 | 1003.2 | 2002.95 | 5.01161 |
| 0.154967 | 1003.2 | 2002.97 | 4.99174 |
| 0.156633 | 1003.21 | 2002.96 | 5.00979 |
| 0.1583 | 1003.2 | 2002.97 | 5.03271 |
| 0.159967 | 1003.19 | 2002.98 | 5.05141 |
| 0.161633 | 1003.2 | 2002.97 | 5.04008 |
| 0.1633 | 1003.2 | 2002.97 | 5.02203 |
| 0.164967 | 1003.2 | 2003 | 5.04755 |
| 0.166633 | 1003.2 | 2002.99 | 5.02807 |
| 0.1683 | 1003.23 | 2002.97 | 5.03752 |
| 0.169967 | 1003.21 | 2002.96 | 5.04222 |
| 0.171633 | 1003.22 | 2002.97 | 5.03485 |
| 0.1733 | 1003.24 | 2002.98 | 5.03851 |
| 0.174967 | 1003.22 | 2002.98 | 5.03888 |
| 0.176633 | 1003.21 | 2002.99 | 5.03609 |
| 0.1783 | 1003.22 | 2002.99 | 5.05022 |
| 0.179967 | 1003.23 | 2003.01 | 5.05088 |
| 0.181633 | 1003.23 | 2003.01 | 5.07161 |
| 0.1833 | 1003.25 | 2002.99 | 5.05662 |
| 0.184967 | 1003.24 | 2003 | 5.0689 |
| 0.186633 | 1003.25 | 2003 | 5.06367 |
| 0.1883 | 1003.23 | 2003.01 | 5.07274 |
| 0.189967 | 1003.25 | 2003 | 5.06655 |
| 0.191633 | 1003.26 | 2003.02 | 5.07767 |
| 0.1933 | 1003.25 | 2003.02 | 5.09573 |
| 0.194967 | 1003.25 | 2003.02 | 5.11082 |
| 0.196633 | 1003.28 | 2003.01 | 5.10739 |
| 0.1983 | 1003.27 | 2003 | 5.12091 |
| 0.199967 | 1003.27 | 2003.02 | 5.09128 |
| 0.201633 | 1003.28 | 2003.02 | 5.11079 |
| 0.2033 | 1003.28 | 2003.04 | 5.12999 |
| 0.204967 | 1003.27 | 2003.04 | 5.12505 |
| 0.206633 | 1003.28 | 2003.02 | 5.12188 |
| 0.2083 | 1003.29 | 2003.04 | 5.10386 |
| 0.209967 | 1003.28 | 2003.05 | 5.11407 |
| 0.211633 | 1003.28 | 2003.05 | 5.10511 |
| 0.2133 | 1003.28 | 2003.05 | 5.12088 |
| 0.214967 | 1003.3 | 2003.06 | 5.12236 |
| 0.216633 | 1003.3 | 2003.06 | 5.11323 |
| 0.2183 | 1003.31 | 2003.05 | 5.12439 |
| 0.219967 | 1003.32 | 2003.05 | 5.12954 |
| 0.221633 | 1003.35 | 2003.04 | 5.12473 |
| 0.2233 | 1003.35 | 2003.06 | 5.12903 |
| 0.224967 | 1003.32 | 2003.05 | 5.13512 |
| 0.226633 | 1003.32 | 2003.07 | 5.13101 |
| 0.2283 | 1003.33 | 2003.07 | 5.13712 |
| 0.229967 | 1003.31 | 2003.08 | 5.14946 |
| 0.231633 | 1003.32 | 2003.08 | 5.14312 |
| 0.2333 | 1003.31 | 2003.06 | 5.14235 |
| 0.234967 | 1003.33 | 2003.08 | 5.14554 |
| 0.236633 | 1003.35 | 2003.09 | 5.16512 |
| 0.2383 | 1003.34 | 2003.08 | 5.14831 |
| 0.239967 | 1003.32 | 2003.08 | 5.15912 |
| 0.241633 | 1003.34 | 2003.1 | 5.15669 |
| 0.2433 | 1003.35 | 2003.1 | 5.15781 |
| 0.244967 | 1003.32 | 2003.09 | 5.18154 |
| 0.246633 | 1003.33 | 2003.1 | 5.17769 |
| 0.2483 | 1003.33 | 2003.1 | 5.1754 |
| 0.249967 | 1003.33 | 2003.1 | 5.17214 |
| 0.251633 | 1003.36 | 2003.11 | 5.18154 |
| 0.2533 | 1003.36 | 2003.11 | 5.1576 |
| 0.254967 | 1003.36 | 2003.13 | 5.19342 |
| 0.256633 | 1003.35 | 2003.1 | 5.20206 |
| 0.2583 | 1003.35 | 2003.11 | 5.1994 |
| 0.259967 | 1003.34 | 2003.14 | 5.23672 |
| 0.261633 | 1003.36 | 2003.12 | 5.24024 |
| 0.2633 | 1003.38 | 2003.12 | 5.22076 |
| 0.264967 | 1003.37 | 2003.13 | 5.21877 |
| 0.266633 | 1003.36 | 2003.13 | 5.21609 |
| 0.2683 | 1003.37 | 2003.13 | 5.21674 |
| 0.269967 | 1003.38 | 2003.15 | 5.20944 |
| 0.271633 | 1003.38 | 2003.14 | 5.21034 |
| 0.2733 | 1003.4 | 2003.13 | 5.21466 |
| 0.274967 | 1003.39 | 2003.14 | 5.24904 |
| 0.276633 | 1003.39 | 2003.15 | 5.26431 |
| 0.2783 | 1003.4 | 2003.14 | 5.24764 |
| 0.279967 | 1003.41 | 2003.15 | 5.24655 |
| 0.281633 | 1003.41 | 2003.15 | 5.24127 |
| 0.2833 | 1003.42 | 2003.15 | 5.24854 |
| 0.284967 | 1003.43 | 2003.14 | 5.23333 |
| 0.286633 | 1003.43 | 2003.15 | 5.24494 |
| 0.2883 | 1003.43 | 2003.16 | 5.26181 |
| 0.289967 | 1003.44 | 2003.17 | 5.24673 |
| 0.291633 | 1003.43 | 2003.17 | 5.26191 |
| 0.2933 | 1003.43 | 2003.17 | 5.25583 |
| 0.294967 | 1003.43 | 2003.18 | 5.27543 |
| 0.296633 | 1003.44 | 2003.17 | 5.26623 |
| 0.2983 | 1003.43 | 2003.19 | 5.27747 |
| 0.299967 | 1003.44 | 2003.18 | 5.24853 |
| 0.301633 | 1003.43 | 2003.19 | 5.27586 |
| 0.3033 | 1003.44 | 2003.19 | 5.27894 |
| 0.304967 | 1003.45 | 2003.19 | 5.29462 |
| 0.306633 | 1003.47 | 2003.19 | 5.28209 |
| 0.3083 | 1003.46 | 2003.18 | 5.28524 |
| 0.309967 | 1003.43 | 2003.2 | 5.28474 |
| 0.311633 | 1003.44 | 2003.2 | 5.29998 |
| 0.3133 | 1003.43 | 2003.21 | 5.29352 |
| 0.314967 | 1003.46 | 2003.19 | 5.29409 |
| 0.316633 | 1003.47 | 2003.2 | 5.29098 |
| 0.3183 | 1003.47 | 2003.2 | 5.28465 |
| 0.319967 | 1003.47 | 2003.2 | 5.31007 |
| 0.321633 | 1003.5 | 2003.22 | 5.28905 |
| 0.3233 | 1003.49 | 2003.23 | 5.30757 |
| 0.324967 | 1003.5 | 2003.23 | 5.30827 |
| 0.326633 | 1003.51 | 2003.22 | 5.33039 |
| 0.3283 | 1003.52 | 2003.21 | 5.34726 |
| 0.329967 | 1003.51 | 2003.22 | 5.32756 |
| 0.331633 | 1003.51 | 2003.24 | 5.34116 |
| 0.3333 | 1003.51 | 2003.23 | 5.33873 |

2. The authors hypothesize that AtlE releases EPA to the environment and this in turn increases lethality of E. faecalis. While it is clear that AtlE and EPA-decorated cell wall fragments play a prominent role during systemic infection in the zebrafish model, the link between the two factors during in vivo infection has not been directly demonstrated. Have you tested if co-injection of WT EPA-decorated cell wall fragments restore lethality of the ∆atlE strain?

We have repeated the complementation of the ∆*atlE* mutant with WT cell wall fragments. Fig. 10 now shows that co-injection of WT EPA-decorated cell wall fragments restore lethality of the mutant.

And conversely, if AtlA and AtlE happen to be functionally redundant, do EPA-decorated cell wall fragments of the ∆atlE strain (released by AtlA) boost virulence of the WT strain?

Our previous work (Salamaga et al., 2017 *PLoS Pathogens*) showed that AtlA contributes to virulence through the control of bacterial cell chain length. This study reveals that the *atlE* mutation has no impact on cell chain length in exponential or stationary phase (Fig. 3). This therefore suggests that the two enzymes contribute to virulence through distinct mechanisms.

For the sake of keeping the message of this paper clear, we would rather not expand too much on the overlapping functions of AtlA and AtlE beyond the evidence provided in Figure 7 investigating protein abundance and activity.

2. At multiple points in the manuscript, the authors claim that the released cell wall fragments act as decoy molecules to inhibit the immune response, however no data are provided to substantiate this interpretation. Experiments to investigate should be performed. For example, previous work in this group has advanced our knowledge on the role of EPA during infection and interaction with macrophages. Do the EPA-decorated cell wall fragments inhibit the host immune system as opposed to the cell wall-attached EPA polymer. Do released EPA fragments protect from phagocytosis and charged antimicrobial peptides? This might help explain why the WT strain that already produces EPA-decorated cell wall fragments is more virulent when exogenous fragments are co-injected. Is it a matter of concentration?
3. We have now demonstrated that EPA is released in culture supernatants, whilst no EPA is detected in *atlE* supernatants (Figure 8). We have also shown that the lack of virulence of the *atlE* mutant is associated with an increased uptake by phagocytes (Figure 9). Antimicrobial assays do not show any impact of the *atlE* mutation (see below). This is expected since the mutant does not show any change in EPA structure (Fig. S6).

**Part III – Minor Issues: Editorial and Data Presentation Modifications**

1. In the response to reviewer 1, in the summary section, the comment about "a previous study showed that soluble peptidoglycan fragments have no impact on bacterial virulence in the zebrafish model of infection" is a misleading statement because the referenced paper refers to *S. aureus*, not *E. faecalis*.

Fair point, we apologise for this omission! We have added a clear reference to the Nature Microbiol paper and indicated that this was *S. aureus*. (p. 21, L.428-429).

2. In the response to reviewer 1, Major issue comment 1, the authors inability to complement on a plasmid due to tetracycline interference can and should be overcome with chromosomal complementation, which is somewhat standard practice in the *E. faecalis* literature.

There is no issue with the complementation, we just omitted this experiment because we felt that this result would not add much. The key result is that the WT releases EPA in supernatants and that this is no longer the case in the *atlE* mutant.

Since reviewer 3 also requested this experiment, we have performed it and added the result in the revised figure 8. The spectra are as expected and the phosphorus NMR is even clearer, probably due to a higher amount of cell walls released in supernatant. This is expected given that the complementation strategy also leads to an overexpression of AtlE (Fig. S5).

3. We note a number of typos in the discussion, so a thorough editing would be useful.

We have checked the discussion and corrected several typos.

Previous minor comments listed here: (we have copied our previous reply)

1. Since AtlE requires EPA decorations to hydrolyse peptidoglycan and AtlE is active during stationary phase, do you know if EPA surface exposure or EPA decorations are more abundant during stationary phase than during exponential phase?

We have added a figure in the revised manuscript to compare the activity of recombinant enzymes on exponential and stationary cell walls and investigated how EPA modulates enzymatic activity. The data indicate that the presence of EPA inhibits the activity of AtlA and confirmed that EPA is essential for AtlE activity. Further investigations are underway do compare the structure of EPA during growth. Answering this question is not trivial and requires complex and time-consuming NMR experiments. We therefore feel that elucidating the structural dynamics of EPA is beyond the scope of this study.

2. Why was it necessary to use the cell wall substrate of a weakened cell wall strain for the zymogram assay in Fig. 1? Similarly, the OG1RF AtlE was able to digest the weakened cell wall of the JH2-2 strain in Figure S2, even though AtlE was later shown to be strain specific. Is this cross-reactivity due to the weak cell wall of this PBP mutant strain?

Zymograms were always carried out with homologous cell walls. For JH2-2, we used a triple *pbp* mutant but for PG1RF extracts, we used wild-type cell walls since we did not have *pbp* mutants in this genetic background. OG1RF zymograms were quite tricky to generate but nevertheless clearly revealed both AtlA and AtlE activities.

3. Fig. 3: why t test instead of ANOVA?

This is a valid point and a mistake we made. A one-way ANOVA is more suitable to compare average values from multiple groups. We have re-analyzed the data using a one-way ANOVA, as suggested; figure 3, its legend and text have been modified accordingly. No major change in our conclusions has been made.

4. L139: typo “altA and altE”

Corrected (p.8, L. 159)

5. L149: typo “led had”

This sentence was modified

6. L306: typo “he injection”

This sentence has been modified in the revised version of the manuscript.

Response to Reviewer #3

**Part I – Summary**

This revised manuscript by Smith et al. describes identification of AtlE, an N-acetylmuramidase active in stationary phase in Enterococcus faecalis. The atlE gene is encoded in the biosynthetic locus for the enterococcal polysaccharide antigen Epa. The authors use a combination of genetics, biochemistry, NMR, and a zebrafish model to show the effect of AtlE on peptidoglycan, release of Epa into supernatants, and on E. faecalis virulence. Overall, this is a nice study that contributes knowledge

The authors have responded to the reviewer comments and revised the manuscript appropriately. I still have a slight concern with the naming convention of *atlE* given the location in the genome but accept the author's rationale for keeping the *atlE* nomenclature. I also am unclear about the rationale for not providing the *atlE* complementation strain in Figure 8 given that several other figures include complementation. However, I think the other data supports the activity and biological importance of AtlE.

As indicated in our response to reviewer 2, we omitted this experiment because we felt that this result would not add much, the main point being to show that the WT releases EPA in supernatants.

Since reviewer 2 also raised an issue with this missing experiment, we have performed it and added the result in the revised figure 8. The spectra are as expected and the phosphorus NMR is even clearer, probably due to a higher amount of cell walls released in supernatant. This is expected given that the complementation strategy also leads to an overexpression of AtlE (Fig. S5).

**Part II – Major Issues: Key Experiments Required for Acceptance**

A key tenet of the study is that AtlE affects virulence by modulating release of Epa. While the study now shows an effect on Epa release in the *atlE* mutant, this is not complemented. Several other figures present complementation data for *atlE*, so I am unclear about the rationale provided about why complementation was not feasible for showing Epa release. If it is not feasible to do the NMR experiments, even a relatively straightforward native gel stained with Alcian blue or Stains-All with polysaccharides precipitated from the supernatant would conclusively show complementation and strengthen the role of AtlE.

See comment above.

The experiment has been carried out and Fig. 8 has been modified accordingly.

**Part III – Minor Issues: Editorial and Data Presentation Modifications**

n/a
